# Direct letters to relatives at risk of hereditary cancer—study protocol for a multi-center randomized controlled trial of healthcare-assisted versus family-mediated risk disclosure at Swedish cancer genetics clinics (DIRECT-study)

**DOI:** 10.1186/s13063-023-07829-5

**Published:** 2023-12-17

**Authors:** Carolina Hawranek, Hans Ehrencrona, Anna Öfverholm, Barbro Numan Hellquist, Anna Rosén

**Affiliations:** 1 Department of Radiation Sciences, Oncology, Umeå University, Umeå, Sweden; 2 Division of Clinical Genetics, Department of Laboratory Medicine, Lund University, Lund, Sweden; 3 Department of Oncology, Institute of Clinical Sciences, Sahlgrenska Academy, University of Gothenburg, Göteborg, Sweden

**Keywords:** Hereditary breast and ovarian cancer, Genetic testing, Lynch syndrome, Randomized controlled trial, Cancer prevention, Risk disclosure

## Abstract

**Background:**

The results of germline genetic testing for hereditary cancer are of importance not only to the patients under investigation but also to their genetic at-risk relatives. Standard care is to encourage the proband (first family member under investigation) to pass on this risk information to the relatives. Previous research suggests that with family-mediated disclosure, only about a third of at-risk relatives contact health care to receive genetic counselling. In some studies, complementing family-mediated risk disclosure with healthcare-assisted risk disclosure almost doubles the uptake of genetic counselling in at-risk relatives. In this study, we evaluate healthcare-assisted direct letters to relatives at risk of hereditary cancer syndromes in a randomized controlled trial.

**Methods:**

Probands are recruited from Swedish outpatient cancer genetics clinics to this two-arm randomized controlled trial. The study recruits probands with either a pathogenic variant in a cancer susceptibility gene (*BRCA1*, *BRCA2*, *PALB2*, *MLH1*, *MSH2*, *MSH6*, *PMS2*) or probands with familial breast and colorectal cancer based on clinical and pedigree criteria. In both arms, probands receive standard care, i.e., are encouraged and supported to pass on information to relatives. In the intervention arm, the proband is also offered to have direct letters sent to the at-risk relatives. The primary outcome measure is the proportion of at-risk relatives contacting a Swedish cancer genetics clinic within 12 months of the proband receiving the test results.

**Discussion:**

This paper describes the protocol of a randomized controlled clinical trial evaluating a healthcare-assisted approach to risk disclosure by offering the probands to send direct letters to their at-risk relatives. The results of this study should be informative in the future development of risk disclosure practices in cancer genetics clinics.

**Trial registration:**

ClinicalTrials.gov. Identifier NCT04197856 (pre-trial registration on December 13, 2019).

Also registered at the website “RCC Cancerstudier i Sverige” as study #86719.

**Supplementary Information:**

The online version contains supplementary material available at 10.1186/s13063-023-07829-5.

## Administrative information

Note: the numbers in curly brackets in this protocol refer to SPIRIT checklist item numbers. The order of the items has been modified to group similar items (see http://www.equator-network.org/reporting-guidelines/spirit-2013-statement-defining-standard-protocol-items-for-clinical-trials/).

**Table:** 

Title {1}	Direct letters to relatives at risk of hereditary cancer—a randomized controlled trial of healthcare-assisted versus family-mediated risk disclosure at cancer genetics clinics in Sweden (DIRECT-study)
Trial registration {2a and 2b}	ClinicalTrials.gov Identifier: NCT04197856 (pre-trial registration). RCC Cancerstudier i Sverige: #86719
Protocol version {3}	This is the first published study protocol in English. Previous instructions in Swedish have been available at all recruiting study sites before the start of the study, and publicly available online at the study website www.umu.se/direct [1] since 2020-03-26.
Funding {4}	The study is funded by - the Swedish Research Council for Health, Work life and Welfare (FORTE), grant 2018-00964, PI Rosén, - the Cancer Research Foundation, grant 2020-1107, PI Rosén, - the Swedish Research Council, grant 2022-02226, PI Rosén. The study also received financial support through the regional agreement between Umeå University and Västerbotten County Council and from the Regional Cancer Centre North. The participating study sites recruiting patients receive a monthly financial support to compensate for expenses associated with the execution of the study. The financial support corresponds to the remuneration for 8 h of work per week for a research nurse.
Author details {5a}	Carolina Hawranek, Department of Radiation Sciences, Umeå University, Umeå Sweden Hans Ehrencrona, Division of Clinical Genetics, Department of Laboratory Medicine, Lund University, Lund, Sweden Anna Öfverholm, Department of Oncology, Institute of Clinical Sciences, Sahlgrenska Academy, University of Gothenburg, Göteborg, Sweden Barbro Numan Hellquist, Department of Radiation Sciences, Umeå University, Umeå, Sweden Anna Rosén, Department of Radiation Sciences, Umeå University, Umeå, Umeå Sweden
Name and contact information for the trial sponsor {5b}	Investigator-initiated trial. Contact person; Anna Rosén, Department of Radiation Sciences, Oncology, Umeå university, SE-901 87 Umeå, Sweden. E-mail: anna.rosen@umu.se.
Role of sponsor {5c}	This is an investigator-initiated trial. The sponsor is the institution of the principal investigator, who has procured independent funding for the study. The funders are not involved in, or have any authority over, study design, data collection, study management, data analysis, interpretation of data, writing of research reports or decisions to submit manuscripts for publication. The funders do require that publication of the research results shall be openly accessible without delay, but this is in line with the wish of the investigators of the study.

## Introduction

### Background and rationale {6a}

Genetic analysis identifying cancer-predisposing gene variants in an individual patient (proband) may prove important also for genetic at-risk relatives (ARRs). Relatives at risk who are informed about the test results can access genetic counselling and predictive testing. Targeted cancer prevention programs are available for confirmed carriers of pathogenic gene variants associated with hereditary breast and ovarian cancer (*BRCA1*, *BRCA2*, *PALB2*) or Lynch syndrome (*MLH1*, *MSH2*, *MSH6*, *PMS2*). These programs include intensified surveillance and risk-reducing surgery reducing cancer incidence and cancer-specific mortality [2–4].

However, the prevention programs in high-risk families are dependent on effective risk disclosure to ARRs to offer eligible individuals an informed choice to undergo genetic counselling and predictive testing and take preventive action or not. The cost-effectiveness of targeted cancer prevention program in high-risk families is linked to the proportion of ARRs who choose to enroll in appropriate preventive care [5].

In most countries, the current standard approach for risk disclosure is to rely on the proband to pass on information to relatives, so called family-mediated risk disclosure. Recently, the first meta-analysis showed that 35% [95% CI, 24 to 48] of ARRs underwent genetic counselling with family-mediated risk disclosure [6]. With healthcare-assisted risk disclosure using direct contact with ARRs, the uptake almost doubled to 63% [95% CI, 49 to 75]. These results suggest direct contact to be a promising complement in risk disclosure practices. However, as most included studies were small and observational, the need for evaluations through randomized controlled trials persists.

In previous research, direct contact between healthcare providers and ARRs has been described as an acceptable route to complement traditional family-mediated risk disclosure pathways [7–10]. Also, patients with personal experience of genetic counselling for hereditary cancer express a desire for a shared proband-healthcare responsibility of informing relatives [11–13].

In the preparations for the trial outlined in this paper, we have conducted both qualitative and quantitative studies and an ethical analysis on hereditary cancer risk disclosure [14–16]. Informed by our preparatory work and in collaboration between representatives of involved study sites, we drafted a study protocol for evaluating direct contact with ARRs at Swedish cancer genetics clinics. We invite probands being offered screening or predictive testing for variants in *BRCA1*, *BRCA2*, *PALB2*, *MLH1*, *MSH2*, *MSH6*, and *PMS2*. The aim of the intervention is to facilitate the dissemination of risk information to ARRs, so they in turn are given the chance to make their own informed choice about genetic counselling. We chose to recruit probands being offered genetic testing for variants associated with adult-onset hereditary breast, ovarian, and colorectal cancer. In the trial, we evaluated risk disclosure to their ARRs after post-test genetic counselling. The study includes families with a pathogenic variant in any of these high-penetrant genes as well as families fulfilling clinical and pedigree-based criteria for familial breast cancer and familial colorectal cancer. Cancer prevention programs for ARRs at risk of these conditions are available and warranted [2, 4]. The intervention includes an offer from healthcare to send direct letters to ARRs as a complement to standard care. The primary outcome measure is the proportion of eligible ARRs who contact a Swedish cancer genetics clinic within 12 months.

### Objectives {7}

#### Research hypothesis

Offering probands direct letters to ARRs as a complement to standard care increases the proportion of ARRs seeking genetic counselling, compared to standard care alone.

#### Study objectives

- *Primary objective*: To determine if offering probands direct letters to eligible ARRs as a complement to standard care is superior to standard care alone
- *Outcome measure*: The proportion of eligible ARRs contacting a Swedish cancer genetics clinic within 12 months of the proband receiving post-test genetic counselling.

The primary outcome will be presented for the prioritized subgroup (participants with a pathogenic variant in *BRCA1*, *BRCA2*, *PALB2*, *MLH1*, *MSH2*, *MSH6*, or *PMS2*) and for all included participants.

- *Secondary objectives*: To determine if offering probands direct letters to eligible ARRs as a complement to standard care is superior to standard care alone among (i) first-degree ARRs and (ii) second-degree, third-degree, or more distant ARRs in families with a pathogenic variant in *BRCA1*, *BRCA2*, *PALB2*, *MLH1*, *MSH2*, *MSH6*, or* PMS2*
- *Outcome measure*: The proportion of eligible ARR contacting a Swedish cancer genetics clinic within 12 months of the proband receiving post-test counselling because the proband have a pathogenic variant in *BRCA1*, *BRCA2*, *PALB2*, *MLH1*, *MSH2*, *MSH6*, or *PMS2*, among (i) first-degree ARRs and (ii) second-degree, third-degree, or more distant ARRs
- To describe acceptance and distribution of direct letters in the intervention group
- *Outcome measure*: Proportion of eligible ARRs to whom the probands allowed a letter to be sent, where contact data allowed distribution of letters, and the letters were collected from the post-office within 12 months of the proband receiving the post-test genetic counselling, stratified by study site, gender, and family diagnosis.

All study outcomes are also presented in Table 1.

**Table 1:** Outcome measures and methods of statistical analysis for primary and secondary outcomes

Outcome	Variable	Description	Methods of analysis
Primary	Proportion of ARRs contacting a cancer genetics clinic	Comparing intervention and control group with respect to proportion of ARRs who have contacted a Swedish cancer genetics clinic within 12 months of the proband receiving post-test genetic counselling from the hereditary cancer investigation	Two-tailed chi-square tests
	Proportion of ARRs contacting a cancer genetics clinic	Comparing intervention and control group with respect to proportion of ARRs who have contacted a Swedish cancer genetics clinic within 12 months of the proband receiving post-test genetic counselling from the hereditary cancer investigation, taking into account study site, gender, age group, and family diagnosis	Logistic regression
Secondary	Proportion of first-degree ARRs contacting a cancer genetics clinic	Comparing intervention and control group with respect to proportion of first-degree ARRs who have contacted a Swedish cancer genetics clinic within 12 months of the proband receiving post-test genetic counselling because the proband is a carrier of a pathogenic variant in BRCA1, BRCA2, PALB2, MLH1, MSH2, MSH6, and PMS2	Two-tailed chi-square tests
	Proportion of distant ARRs contacting a cancer genetics clinic	Comparing intervention and control group with respect to proportion of second-degree, third-degree, or more distant ARRs who have contacted a Swedish cancer genetics clinic within 12 months of the proband receiving post-test genetic counselling because the proband is a carrier of a pathogenic variant in BRCA1, BRCA2, PALB2, MLH1, MSH2, MSH6, and PMS2	Two-tailed chi-square tests
	Acceptance of the intervention	Proportion of ARRs who the probands allowed contact with, stratified by study site, gender, and family diagnosis	Two-tailed chi-square tests
	Distribution of direct letters	Proportion of ARRs who the probands allowed contact with and where contact data allowed distribution of the direct letter, stratified by study site, gender, and family diagnosis	Two-tailed chi-square tests
	Collection of direct letters	Proportion of ARRs who the probands allowed contact with, where contact data allowed distribution of letters, and the letters were collected from the post-office within 12 months of the proband receiving post-test genetic counselling, stratified by study site, gender, and family diagnosis	Two-tailed chi-square tests

### Trial design {8}

The trial design is a pragmatic, multi-center, parallel assignment, balanced ratio, open-label, randomized, controlled superiority trial in Sweden.

## Methods: participants, interventions, and outcomes

### Study setting {9}

**Fig. 1:**
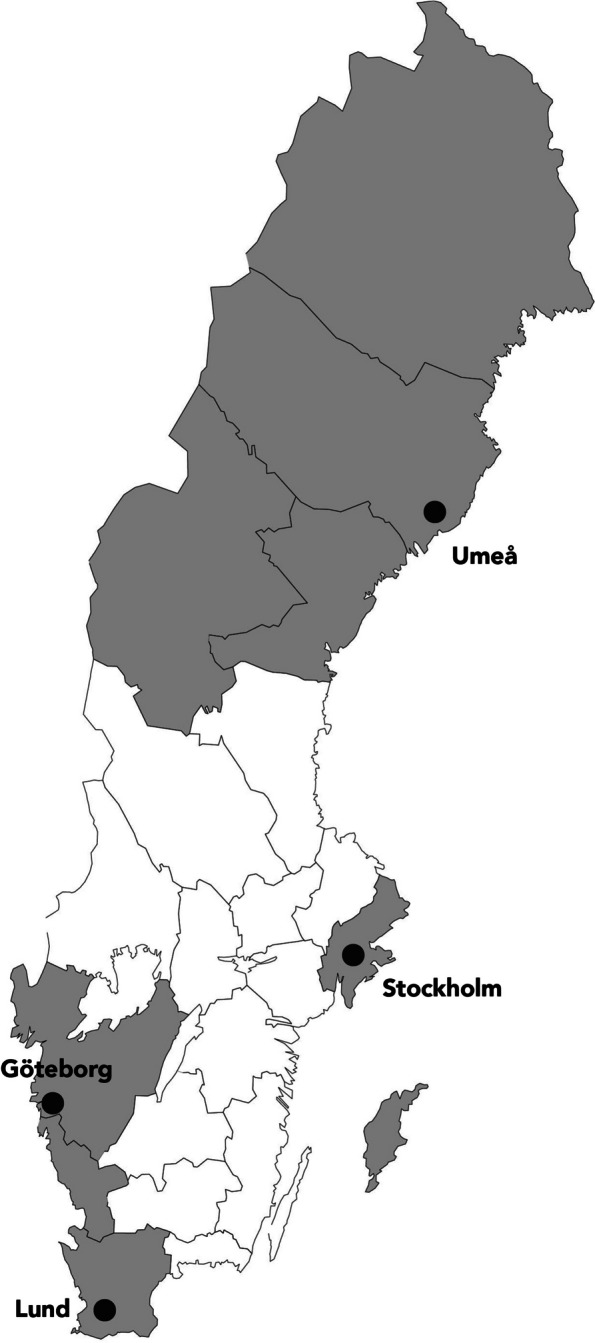
Map of included study sites and their catchment area

Probands are recruited from outpatient cancer genetics clinics at the following university hospitals in Sweden: University Hospital of Umeå, Karolinska University Hospital, Sahlgrenska University Hospital, and Skåne University hospital (Fig. 1).

### Eligibility criteria {10}

#### Eligibility criteria for participants

The recruitment basis is adult individuals (18 years and older), fulfilling clinical criteria for genetic screening or targeted carrier testing for pathogenic variants in any of the following genes: *BRCA1*, *BRCA2*, *PALB2*, *MLH1*, *MSH2*, *MSH6*, *PMS2*.

Inclusion criteria:

1. Proband offered cancer genetic investigation and post-test genetic counselling for hereditary breast, ovarian, or colorectal cancer
2. Written consent to participate in the study
3. Belonging to a family with either (a) familial breast cancer, (b) familial colorectal cancer, or (c) a pathogenic variant in *PALB2*, *BRCA1*, *BRCA2* (hereditary breast and ovarian cancer), *MLH1*, *MSH2*, *MSH6*, or *PMS2* (Lynch syndrome)
4. Having at least one eligible ARR (family member deemed to be an ARR recommended genetic counselling within a year)

Exclusion criteria:

1. Inability to convey personal opinions and preferences or
2. No eligible ARRs living in Sweden

**Fig. 2:**
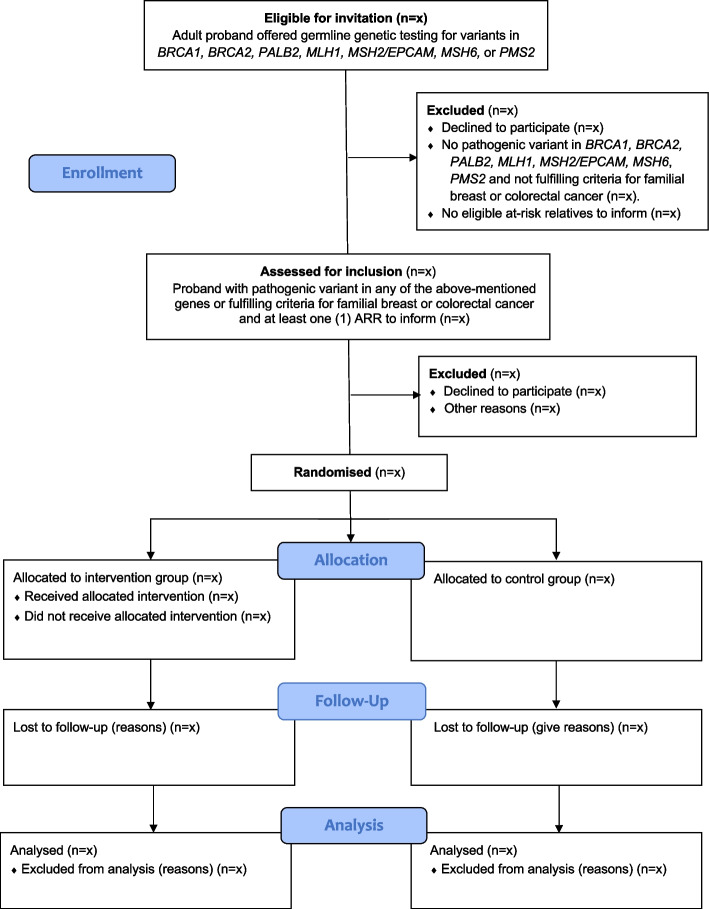
Flow chart of the DIRECT-study

The CONSORT flow diagram is found in Fig. 2.

#### Eligibility criteria for study sites

Inclusion criteria

1. Outpatient cancer genetics clinics in Sweden offering post-test genetic counselling
2. Interest in the study and offering dedicated resources to implement the study protocol
3. Participation in preparatory meetings and contribution to the development of the study protocol
4. Assigning a registered nurse or genetic counsellor to act as local study coordinator
5. Assigning a specialist in clinical genetics as responsible for clinical and patient safety

Exclusion criteria

1. Audit revealing unfulfilled pilot/progress criteria

### Who will take informed consent? {26a}

Health care professionals (HCPs) associated with the study (registered nurses, genetic counsellors, or physicians) will give oral information about the study to potential participants. Written participant information and consent form are given or sent by mail to the participant.

If counselling is given by phone or online appointment, written information is sometimes given before oral information.

Both routines (oral first, written second or vice versa) have been approved by the ethical authorities beforehand.

Each participant signs the informed consent prior to the initiation of any study-related procedures (distribution of questionnaires or sending direct letters to ARRs).

### Additional consent provisions for collection and use of participant data and biological specimens {26b}

NA. This trial does not involve collecting biological specimens for storage.

## Interventions

### Explanation for the choice of comparators {6b}

The comparator is family-mediated risk disclosure to ARRs. All probands receive genetic counselling according to current clinical practice (standard care), including information from an HCP who encourages family-mediated disclosure of hereditary cancer risk to ARRs.

The additional component in the control group is the clinical documentation of eligible ARRs for each study participant. The HCP lists the ARRs in collaboration with the proband, who is asked to provide contact details to eligible ARRs. This component is not standard care.

The listing of ARRs will be completed during the post-test counselling session and/or complemented by a follow-up telephone call from the local genetic counsellor or the study coordinator. Detailed listing of ARRs has been an interventional component in previous studies attempting to increase proband-mediated risk disclosure to ARRs [17]. The effect of this procedure has not conclusively proven to impact on uptake, and it is not possible to distinguish the effect of detailed listing of relatives from other parts of the interventions under study. In an RCT evaluating an additional genetic counselling session by telephone (including detailing ARRs), the intervention did not have any effect on number of ARRs contacting a genetic clinic. In another retrospective cohort study with a multicomponent intervention including enhanced genetic counselling with pedigree discussion, the intervention led to a significantly increased the number of ARRs contacting the genetic services [18].

Hence, we acknowledge that the listing procedure conducted in both the intervention and control arm could potentially impact the outcome. However, this step of detailing ARRs is essential for collecting the study-related outcome data and was equally performed in both groups.

### Intervention description {11a}

Participants in the intervention group receive the same standard care and listing of eligible ARR by the HCP as the control group participants outlined in the “Explanation for the choice of comparators {6b}” section. In addition, study participants allocated to the intervention arm are offered the option of the HCP sending a direct letter to their ARRs deemed to be recommended genetic counselling within a year. He or she then provides or deny approval for each ARR to be contacted by direct letter. The direct letters are sent by HCP from the local cancer genetics clinic to those ARRs who the participant/proband approves contact with. However, if an eligible ARR contact the same local cancer genetics clinic before the distribution of letter, a letter is not sent to that specific ARR.

The direct letter informs the ARR about the conducted genetic investigation of hereditary cancer and possible implications for them and their family (see templates of letters in Additional file 4). The letters are designed to be brief and phrased in a general way but still tailored to the family they concern. Contact details to the nearest cancer genetics clinic are included on all letters. The letters are sent about 1 month after the participant has received the post-test genetic counselling, but in some cases, the time is prolonged according to the participant’s preferences. Distribution of letters is paused before national holidays or during the summer vacations to reduce the risk of delayed contact with an available genetics HCP.

The letters are sent with registered mail where the recipients/ARRs need proof of identity to retrieve the letter. The cancer genetics clinic will receive the letter in return should the addressee fail to collect the letter within a 2 weeks’ time.

### Criteria for discontinuing or modifying allocated interventions {11b}

The intervention is *an offer* of sending direct letters to the probands’ ARRs, and the participant may opt out or modify the intervention and still remain in the trial. Hence, even if the proband modify to whom the direct letters will be sent and the timing of the distribution of letters, data for follow-up will be retained.

### Strategies to improve adherence to interventions {11c}

NA. No additional strategies are employed for improving adherence, as the intervention is an offer the participant can either accept or refuse.

### Relevant concomitant care permitted or prohibited during the trial {11d}

NA. All clinical care, including different local strategies for encouraging participants to contact their relatives, are permitted.

### Provisions for post-trial care {30}

All participants, and their relatives, may contact their local cancer genetics clinic and receive counselling and support before, during, and after the study.

### Outcomes {12}

The study’s primary and secondary outcomes are presented in Table 1.

Time point for all outcomes (*T*_3_) is 12 months after the time when the proband receives post-test genetic counselling about the hereditary cancer investigation and its implications for family members (*T*_0_).

The denominator in the primary outcome is defined as those ARRs that are deemed to be recommended genetic counselling within 12 months according to the involved HCP.

The nominator in the primary outcome is defined as those ARRs that have any interaction with a Swedish cancer genetics clinic either by phone, electronic communication, or physical meeting between *T*_0_ and *T*_3_.

This data is collected by the local study coordinator by checking local patient data registries and/or asking the other national collaborating units if they have had any registered contact with ARR within 12 months after *T*_0_. The outcome for each proband is then reported to the study database as plain numbers, with details only on uptake of contact (number of ARR in denominator and nominator), gender (number of female and male ARRs), and relationship to the patient (number of first-degree ARRs and other ARRs).

### Rationale for the choice of primary outcome

The primary outcome, ARRs’ contact with a Swedish cancer genetics clinic, offers the possibility of identifying any interaction with a genetic HCP, thus revealing that enough information has been transmitted to the ARR to be able to get in contact with a cancer genetics unit, if they wished to do so. We use this measure as a proxy for genetic counselling, as long-term clinical experience from the involved study sites implies that this measure is closely related to the uptake of genetic counselling. We are aware that the number of ARRs’ contacting a clinic is lower than the number of ARRs informed in the first place, as an informed relative may decide not to contact the clinic during the follow-up period. In Sweden, the vast majority of ARRs pursue genetic counselling after their first contact. Another reason for the choice of the primary outcome is its alignment with previous [6] and ongoing [19] evaluations of risk disclosure interventions that have also utilized ARR’s contact with a cancer genetics clinic as an outcome measure. Thus, our data will be possible to include in future meta-analyses focused on risk disclosure.

An important ethical concern in conducting this study is the fact that it is the probands, not the ARRs, who consent to the study and is allocated to intervention or control group, while the outcome relies on information regarding the actions of the ARRs. To address the potential risk of compromising privacy and/or discomfort for the ARRs, we adopt a cautious approach in all aspects of the data collection. Regarding the ARRs, we only collect data on contact with a cancer genetics clinic within the time frame from *T*_0_ to *T*_3_ (yes/no), gender (female/male), and degree of relationship (first-degree/other).

During the study period, updated Swedish national guidelines have introduced mainstream testing of breast and ovarian cancer patients and revised clinical criteria for familial CRC. Consequently, there has been a clear decrease in the number of individuals receiving the diagnoses familial breast cancer and familial CRC. Meanwhile, there has been a scientific debate on prioritizing finding carriers of PV in high-risk genes over offering intensified surveillance to individuals with familial breast cancer and familial CRC. This led us to the decision to prioritize reporting on the patient group with a pathogenic variant in BRCA1, BRCA2, PALB2, MLH1, MSH2, MSH6, or PMS2. This patient group is the target in both previous and ongoing trials evaluating risk disclosure, and thus, this data too will be possible to include in future meta-analyses.

### Rationale for the choice of secondary outcomes

 Previous research shows that the relative’s degree of relationship to the proband impact on uptake [6], and therefore, we will perform subgroup analysis of contact with cancer genetic clinics among first-degree relatives and others (second-degree or more).

To further investigate the upstream decisions enabling direct contact with ARRs, our chosen secondary measures include outcomes which reveal the step-wise process following an offer of a direct letter to ARRs, the probands’ acceptability to inform ARRs by direct letter, the availability of contact details, the distribution of the letter, and the actual delivery of the direct letter to the ARRs.

### Participant timeline {13}

Participants enter the study at time *T*_0_ and receive post-test genetic counselling including listing of their eligible ARRs (Table 2). Approximately 1 month later (*T*_1_), direct letters are sent to eligible ARRs in the intervention group. Twelve months after *T*_0_, the main outcome is summarized (*T*_3_).

**Table 2:** Participant timeline

**Timepoint**	**Enrolment**	**Allocation**	**Post-allocation**		**Close-out**
	− *T*_1_	*T*_0_	*T*_1_	*T*_2_	*T*_3_
Time (in months)	− 3 to 0	0	1	6	12
**Enrolment**					
Eligibility screening	x				
Patient information	x				
Informed consent	x				
Inclusion/inclusion criteria	x				
**Allocation**					
Intervention		x	x		
Control		x			
**Assessments**					
**Screening log**					
Gender	x				
Year of birth	x				
Hereditary cancer family diagnosis (yes/no)	x				
Uninformed eligible ARR (yes/no)	x				
**CRF 1**					
Cancer diagnosis or relapse within a year (yes/no)		x			
Verification of inclusion criteria		x			
**CRF 2**					
Listing of eligible ARRs (stored locally, not shared with the study coordinating center)		x			
**CRF 3**					
Proportion of eligible ARRs contacting a Swedish cancer genetics clinic within 12 months					x
**Questionnaires**					
Participant-reported outcomes		x		x	

Questionnaires on participant-reported outcomes are sent to the participants at time *T*_0_ and 6 months after a second questionnaire is sent (*T*_2_). For more information on these questionnaires, see the “Discussion” section.

### Sample size {14}

In 2019, there was no available published data on the uptake of genetic counselling after family-mediated risk disclosure in Sweden. Due to this lack of certainty, we decided to make the sample size calculations based on the assumption that each proband has 4 ARRs in average and the uptake in the control group is 50%. We wanted to be able to detect if at least one more ARR in every second family contacted a cancer genetics clinic in one of the study arms, i.e.., 5 out of 8 ARRs. Based on this, we determined that the study needed the power to detect a difference of 12.5 percentage units (62.5% in intervention, 50% in control group). To detect this difference with a power of 0.8 and a two-sided 5% significance level required 490 listed ARRs (half in each study group). The power analysis was performed in R version 4.2.1 using the pwr package. Effect was defined as Cohens *h* = 2*asin(sqrt(p1))-2*asin(sqrt(p2)) where p1 = 0.625 and p2 = 0.50.

To allow for subgroup analyses, the recruitment target was set to 600 ARRs. During the study period, clinical guidelines in Sweden changed, putting less focus on familial cancer and more on predictive testing. To adapt to this change, the initial recruitment target of 600 ARRs in total was adopted to 490 ARRs in the most prioritized subgroup, i.e., families with a pathogenic variant identified in a high-risk gene (hereditary breast and ovarian cancer and Lynch syndrome: BRCA1, BRCA2, PALB2, MSH2, MSH6, MLH1, or PMS2).

### Recruitment {15}

In 2018–2019, we reached out to the management and physicians at all Swedish cancer genetics clinics situated within university hospitals, extending invitations for their participation in the study. Among the six clinics approached, four successfully underwent training for the study and commenced patient recruitment in 2020.

At each study site, a local study coordinator is responsible for patient recruitment, but HCPs associated with the study (physicians, registered nurses, and genetic counsellors) are involved in informing potential participants about the study. The local study coordinator oversees the patient recruitment and receives part-time salary for study-related tasks.

HCPs recruit patients attending the participating study sites through a personal invitation. The recruitment basis for the study consists of adults fulfilling clinical criteria for genetic screening or targeted carrier testing for a pathogenic variant in any of the following genes: BRCA1, BRCA2, PALB2, MLH1, MSH2, MSH6, or PMS2. Thus, all patients attending the involved study sites who are eligible for such genetic testing could be considered for an invitation to the study.

The majority of potential participants are approached with study information at pretest counselling. This strategy is necessary to allow for sufficient time for patient recruitment before treatment allocation and before the patients receive post-test counselling. A consequence of this strategy is that most of the potential participants will have a negative test result and not fulfil inclusion criteria for the study.

During the study period, the implementation of so-called mainstream testing of patients with newly diagnosed breast or ovarian cancer led to a new category of patients that already had received their test results when being referred to the cancer genetics clinics for counselling. These patients often fulfill inclusion criteria, and to safeguard the patient recruitment of also these patients, they receive oral and written information about the study directly after being referred to the cancer genetic clinic but before the post-test counselling.

Enrolment started in February 2020 at the University Hospital of Umeå, Sahlgrenska University Hospital, and Skåne University hospital. The patient recruitment at these sites have continued at a slow, but steady rate throughout the study period. Due to different workflows between study sites and over time, recruitment strategies have been adjusted to local routines and new situations (like remote patient contact during the COVID-19 pandemic). The study site Karolinska University hospital recruited patients from August 2020 to November 2021, but on 15 November 2021, the study research team decided to stop further patient recruitment due to poor participant recruitment rate and difficulties in implementing the study protocol.

Assuming 4 ARRs per participant, we would be able to reach the recruitment target of 600 ARRs by including 150 participants. When planning the study, we estimated that the participating study sites would see around 700 patients meeting the inclusion criteria each year, and a modest assumption was that about half of them would accept the invitation allowing us to close the study within 1 year. However, given the commonly encountered recruitment difficulties in clinical trials in general, we planned for participant recruitment in 2 years. With four sites, this equals a recruitment rate of 1.5 participants per site and month. Due to the COVID-19 pandemic, low staff resources, change in guidelines resulting in fewer patients fulfilling criteria of familial colorectal cancer, and mainstream testing leading to less influx of patients with familial breast cancer, we have extended the recruitment period until December 31, 2023 (this amendment was approved by the Swedish Ethical Review Authority). Based on preliminary recruitment data, we expect that the inclusion target will be reached at this date.

## Assignment of interventions: allocation

### Sequence generation {16a}

Participants are randomized at a ratio of 1:1, stratified by gender, age group, and family diagnoses at each site. Site-specific pre-determined random number sequences are used for randomization, accessed through a randomization instrument prepared by the study statistician.

### Randomization instrument

The randomization instrument was built in excel in the form of a workbook that consist of several worksheets. Each study site only had access to the workbook for that site. In the primary worksheet of the workbooks, the study coordinator enters the participant’s gender, age group, and family diagnosis and receives the study group allocation. There are underlying worksheets, one for each combination of gender, age group, and family diagnosis, with random sequences of 1:s and 2:s that determine if the individual is allocated to the control or study group. The sequences were generated using the function RANDBETWEEN, and there is one sequence for each combination of gender, age group, and family diagnosis. When an individual’s gender, age, and family diagnosis are entered on the primary worksheet, an excel formula fetches the study group allocation from the corresponding underlying sheet.

### Concealment mechanism {16b}

Allocation concealment is ensured as the local study coordinator access to the randomization instrument is limited to entering the participant’s gender, age group, and family diagnosis and thereafter receiving the study group allocation. The random number sequence is automatically accessed “under the hood” within the instrument and never shown to the local study coordinator. Thus, HCPs, local study coordinators, and participants are unaware of study group allocation before randomization and cannot predict allocation based on the previous sequence.

### Implementation {16c}

Enrolment of participants is made by HCP at the involved study sites, often after consulting the local study coordinator. At inclusion, the local study coordinator controls inclusion and exclusion criteria and uses the randomization instrument to allocate participants to the intervention or control group.

## Assignment of interventions: blinding

### Who will be blinded {17a}

Since the intervention in this trial is an offer of sending physical direct letters to ARR, neither the HCP nor the study participant can be blinded to the allocation. However, the final data analysis will be performed by a statistician blinded to the study arm allocations and subgroups.

### Procedure for unblinding if needed {17b}

NA. This is an open-label trial.

## Data collection and management

### Plans for assessment and collection of outcomes {18a}

Data for assessment of outcomes is collected with case report forms (CRFs) and questionnaires (see Additional files 2 and 5).

### Case report forms

- CRF1: Research subject data (age, gender, study site, study group, family diagnosis, inclusion date)
- CRF2: Family follow-up data (stored locally) with a list of ARRs recommended genetic counselling within a year. The list is documented in collaboration between an HCP and the proband
- CRF3: Outcome data (e.g., total number of ARRs, number of ARRs who could not be identified or where contact details were not sufficient, total number of ARRs who contacted a Swedish cancer genetics clinic within 12 months, outcome assessment date) and additional outcome data for the intervention group (e.g., number of relatives who the proband consented to contact by direct letter, number of relatives who had already contacted a clinic, number of registered letters which were actually sent, number of registered letters which were collected at the post office)

To promote recruitment and data quality, the local study coordinators receive training and support during the participant enrolment process in the form of the following:

- Detailed study protocol in Swedish, both in print and digital, including a checklist showing all study related actions. The protocol has been developed in close collaboration with participating clinics to account for variations in practice
- Regular online meetings chaired by the coordinating center for questions and sharing of experiences. These meetings were held weekly the first 2 years (2020–2021) and then twice a month from 2022 and onwards
- A study support help line with direct telephone access to the coordinating center, where questions regarding the study protocol application could be addressed on short notice
- Allocated time for HCP at each study site to conduct study-related tasks, financed by the principal investigator
- HCPs at the involved study sites receive regular information about the study in recurrently published newsletters and seminars conducted by the coordinating center

To monitor data quality, inclusion criteria of newly recruited study participants are continually re-evaluated by the study coordinating center in discussion with local study coordinators. If participants are found not to meet inclusion criteria at re-evaluation, these subjects are excluded.

In addition, the denominator in the primary outcome (total number of ARRs) will undergo a second opinion assessment by an independent genetic HCP.

### Questionnaires

We administrated questionnaires on participant-reported outcomes to all participants in both study arms at two time points. The questionnaires include validated instruments measuring generic health-related quality of life (RAND36), anxiety (State-Trait Anxiety Inventory, STAI), and cancer worry (CWS). The RAND36 includes eight dimensions: physical functioning, role limitations caused by physical problems, bodily pain, general health, vitality/energy/fatigue, social functioning, mental health/emotional well-being, and role limitations caused by emotional problems. The State-Trait Anxiety Inventory (STAI) consists of two 20-items subscales; the state subscale (STAI-S) assesses current level of anxiety and the trait subscale (STAI-T) addresses innate and relatively stable personal tendencies to experience anxiety symptoms [20]. The CWS is an eight-item scale measuring frequency and severity of cancer worry as well as impact on mood and daily functioning [21]. Swedish norm data is available for the instruments [22–24]. Repeated questionnaires enable us to evaluate changes over time and differences between groups.

### Plans to promote participant retention and complete follow-up {18b}

We do not have any retention strategies, as information about total number of ARRs per study participant is collected at the time of inclusion, and completeness of follow-up is thereafter not dependent on the participant.

Participants who drop out from the study, for any reason, will not be included in final analyses.

### Data management {19}

Study-specific data is documented on printed paper CRFs at each local study site. The completed CRFs are sent by post to the coordinating center where all CRFs are reviewed before data is manually entered in a study-specific database. Once digitalized, original CRFs are archived. To mitigate data entry errors, a second investigator will review all database entries and make sure they are consistent with the archived CRFs.

The database is stored in a study-specific folder accessed only by research team members formally pre-approved by the principal investigator. The study-specific folder has automated back-ups ensuring possibilities to retrieve data in the case of accidental loss.

During the data collection, all study-related documents (CRFs and consent forms) will be stored securely in facilities with restricted access at the involved cancer genetics clinics. Once the documents arrive to the coordinating center, it is stored at the archive at the Cancer genetics clinic, RCC Norr, Norrlands universitetssjukhus, Umeå. After completion of the study, the documents will be archived in a dedicated secure archive at the Umeå University, Sweden. Final storage and archiving of the electronic data will take place in a dedicated archive at Umeå University electronic file location, on local servers with high safety standards for sensitive personal data. Collected data will be stored for 15 years as required by the Swedish archive law (SFS 1990:782).

For full data management procedures, see the study’s data management plan accessible at the study web page [1].

### Confidentiality {27}

All personal and sensitive data managed in the project is handled according to the EU general data protection act (GDPR) and Swedish patient safety standards (the Swedish law of Secrecy and Public Access). This means that personal and sensitive information of the enrolled participants will be handled according to the Swedish patient safety standards and good clinical practice. Data collection and handling has been pre-approved by the national Ethical Review Authority and will be carried out with the legal basis of public interest.

Digital data collected in the study is stored in a study-specific folder that can only be accessed by team members approved by the principal investigator. Access to this folder requires unique identifier log-in with multi-factor authentication.

Study-related paper-based documentation (CRFs and consent forms) is kept at each study clinic with restricted access exclusively to eligible HCPs. Only pre-defined data points are forwarded to the study-specific database through mailing CRFs to the coordinating center. At the coordinating center, this data is only accessed by co-workers authorized by the principal investigator.

This study will not collect any personal data on the participants’ ARRs. This data, collected in CRF2, will only be handled at the local study site and not shared with the coordinating center. Instead, compiled data on primary and secondary outcome is collected in CRF3 and reported to the coordinating center as plain numbers.

At each study site, the local study coordinator logs data on potential participants. This local screening log does not contain any personal or sensitive data. Compiled data from the local logs are transferred securely to a protected intermediate file location and then imported to the database at regular time intervals.

### Plans for collection, laboratory evaluation, and storage of biological specimens for genetic or molecular analysis in this trial/future use {33}

NA. No biological specimens will be collected in this trial.

## Statistical methods

### Statistical methods for primary and secondary outcomes {20a}

The study’s primary and secondary outcomes, and the statistical methods with which they will be analyzed, are presented in the statistical analysis plan (Table 1).

The primary outcome will be analyzed with chi-square test, and in logistic regression models, both univariable and multivariable. The multivariable model will be adjusted for the stratification variables, i.e., study site, gender, age group, and family diagnosis.

The primary outcome will be presented for the prioritized subgroup (participants with a pathogenic variant in *BRCA1*, *BRCA2*, *PALB2*, *MLH1*, *MSH2*, *MSH6* or *PMS2*) and for all included participants.

All secondary outcomes with be analyzed with chi-square test.

### Interim analyses {21b}

This is a low-risk intervention. However, if the evaluation of any progress criterium is categorized as “alert,” this may trigger termination of the trial (see Additional file 3).

### Methods for additional analyses (e.g., subgroup analyses) {20b}

#### Subgroup and adjusted analyses of the primary and secondary outcomes

We plan to conduct subgroup analyses for different gender (male vs female) and degree of relationship (first degree vs other).

As previously mentioned, we also plan to perform adjusted analysis taking into account the variables used in the allocation process (gender (male vs female), study site (Umeå, Göteborg, Lund), age group (0–50 years vs 50–99 years). and family diagnosis.

#### Analysis of questionnaire data

We will conduct analyses for differences in probands’ health-related quality of life (RAND-36), anxiety (STAI), and cancer worry (CWS) between the different study arms on data from the 6 months follow-up questionnaires, adjusted for baseline values. RAND-36 scores and compositive scores and STAI scores will be presented as means with standard deviation. CWS will be presented as proportions with high and low CWS-score and means with standard deviation. Differences in mean will be tested with *t*-test, and differences in proportion will be tested with chi-square test.

### Methods in analysis to handle protocol non-adherence and any statistical methods to handle missing data {20c}

We will apply intention to treat principle but will also perform per-protocol sensitivity analysis. In this study, eligible ARRs with insufficient contact details for follow-up are included in the intention to treat analysis but excluded from the per-protocol analysis.

### Plans to give access to the full protocol, participant-level data, and statistical code {31c}

This study protocol will be available open access. All previous instructions in Swedish have been publicly available online at the study webpage [15] since 2020 March 26. Individual participant-level data is not possible to disclose due to confidentiality requirements, but access to aggregated data and statistical code may be granted upon reasonable request to the principal investigator.

## Oversight and monitoring

### Composition of the coordinating center and trial steering committee {5d}

The study is coordinated by the principal investigator, employed at Region Västerbotten and affiliated to the Umeå University. The larger study research team includes researchers in nursing, ethics, qualitative and quantitative methodology, oncology, and clinical genetics, but a formal steering committee was not established for this trial.

### Composition of the data monitoring committee, its role and reporting structure {21a}

The intervention is considered as low risk for adverse events. The trial has therefore not appointed an external data monitoring committee.

### Adverse event reporting and harms {22}

The nature of the intervention is such that the main concern about harm is ARRs’ negative psychological reactions to being approached by unsolicited health-related information. The coordinating center collects and documents if HCPs have noted any psychological reaction of the study in participants and/or their ARRs. This data is collected through the regular meetings with the local study coordinators. These events do occur at times in standard care as well, and thus, each clinic involved have established routines and available care if needed.

### Frequency and plans for auditing trial conduct {23}

Formal auditing at each study sites is performed at least annually. The local study coordinator contributes with data to the audit. The first step is completion of a structured report of set progress criteria designed to follow-up protocol adherence and practical implementation at each study site (see Additional file 3). Secondly, an audit interview is performed to elaborate on progress, difficulties, and any uncertainties regarding the study procedures. Audit protocols are documented and saved in the study-specific folder. The audits are conducted by a member of the research team.

### Plans for communicating important protocol amendments to relevant parties (e.g., trial participants, ethical committees) {25}

Approval from the Swedish Ethical Review Authority is sought before any amendment of the protocol. Changes or modifications are documented by the study coordinator team and public study material at the project website and at ClinicalTrials.gov are updated accordingly.

### Dissemination plans {31a}

All scientific publications from this project will be available in open access channels. The study has an official and public website [1] available in both English and Swedish with contact information and relevant official documents. Links to media coverage about the study are collected on the study website to increase reach and public engagement.

Trial participants are informed by HCPs at the participating study sites, but also receive written information with full contact details to all study sites and the study office, with a dedicated telephone number and email, to which all can call and ask any questions about the study at any time.

## Discussion

Genetic testing for hereditary cancer often has implications not only for the individual patient but also for his or her genetic relatives. Current practice in Sweden and most other countries is to encourage the patient to inform his or her relatives. Studies indicate that only a third of ARRs contact health care to receive genetic counselling with this practice.

The starting point for this project is this clinical situation where the interests and duties of the probands, ARRs, and genetic HCPs may be unclear or come into conflict. Our study seeks to mitigate this ethical dilemma by evaluating a collaborative approach with an offer of direct contact between HCP and the ARRs. As long as HCPs act with the explicit consent of the proband, both patient integrity and current legal frameworks are respected.

To our knowledge, this is the first European clinical RCT evaluating the effectiveness of direct letters about hereditary cancer risk to ARRs. In our study, we include patients from families with hereditary risk of breast, ovarian, or colorectal cancer for which there is available evidence-based surveillance programs. We include both probands with a pathogenic variant in BRCA1, BRCA2, PALB2, MLH1, MSH2, MSH6, or PMS2 and probands with pedigree-based estimation of hereditary risk for breast and colorectal cancer. The latter, so called familial cancer diagnoses, have historically constituted a majority of patients at the cancer genetic clinics in Sweden. However, in the last years, and during recruitment to this study, there has been a significant reduction in the number of probands receiving these diagnoses. This is explained by revised referral and diagnostic criteria in national guidelines and a shift towards mainstream clinical genetic testing at time of cancer diagnosis. As described above, this context change prompted the adaptation of our strategy for completion of the study. We are now striving to reach the full recruitment target for probands with a pathogenic variant in the above-mentioned genes.

In parallel with the ongoing RCT, we explore the interventional components of the procedures tested and acceptability among probands and ARRs as well as monitoring of proband-reported outcomes. Proband experiences of participation in the study has been explored through interviews in both study arms (manuscript under review). Questionnaires on proband-reported outcomes are administrated to all participants in both study arms at two time points. They include validated instruments measuring generic health-related quality of life (RAND36), anxiety (State-Trait Anxiety Inventory, STAI), and cancer worry (CWS). Repeated questionnaires enable us to evaluate changes over time and differences between groups. Another ongoing auxiliary study involves ARRs that have received a direct letter. Although the RCT is formally approved by the Swedish ethical review authority, it may still be perceived as ethically controversial, as it is the proband that approves of participation in the study, while the unsolicited direct letters are distributed to the probands’ ARRs. Therefore, a sample of ARRs that have received a direct letter are invited to interviews upon contact with the cancer genetics clinic. It remains to be seen how the ARRs in this study perceive the reception of a direct letter, but there is some evidence available from research on the hereditary colorectal cancer registry in Denmark which has sent unsolicited letters with an invitation to genetic counselling since 1997 directly to relatives in high-risk families. A follow-up study of this practice showed that support for direct letters was expressed by 78% of family members and 90% preferred a letter to no information [8]. Two thirds of respondents also preferred healthcare to be the source of information rather than a distant relative. No adverse psychological effects were identified.

An unresolved dilemma in the control group (and in current practice) is the potential emotional stress and missed opportunities for prevention among ARRs who may be left without information and later find out about their risk. This is also a rationale for conducting this study. Another limitation of addressing the issue of unreached relatives through a research study is that of potential selection bias, where those negative towards risk disclosure may choose not to participate in the study.

The long-term implication of this research has the potential to contribute to relevant clinical guidelines at cancer genetics clinics. Results from this study may also be relevant for other health care settings, where patients are offered germline testing for hereditary conditions.

## Trial status

Patient recruitment started on 6 February 2020. This is the first protocol (version 1.0) in English. The Swedish protocol (“Praktiskt genomförande” 2.8, last updated 2022 September 22) has been publicly available at the study website since the start of the study. Recruitment is estimated to be completed approximately 2023 December 31.

### Supplementary Information

**Additional file 1.** Patient informed consent.

**Additional file 2.** Case report forms.

**Additional file 3.** Progress criteria and study site audit.

**Additional file 4.** Letter templates.

**Additional file 5.** Questionnaires.

## Data Availability

Access to aggregated data may be granted upon reasonable request to the principal investigator.
