# Supplementary material for: Direct letters to relatives at risk of hereditary cancer—study protocol for a multi-center randomized controlled trial of healthcare-assisted versus family-mediated risk disclosure at Swedish cancer genetics clinics (DIRECT-study)
Source: Trials. 2023 Dec 17;24:810. doi: 10.1186/s13063-023-07829-5 (PMC10726564; doi:10.1186/s13063-023-07829-5)
Supplement: Supplementary file 1 — Additional file 1. Patient informed consent. [file 13063_2023_7829_MOESM1_ESM.pdf]

Information
about the DIRECT study

(Two copies, one to be kept and one to be returned)

Information about the DIRECT study

What we are researching and why we are asking you to participate

We who work at cancer genetics clinics counsel families with aggregation of cancer cases where we suspect a hereditary cause. In Sweden there are six oncogenetic clinics and we have a close collaboration between us.

You have received this letter since you are completing a cancer genetic investigation. The investigation’s aim is to evaluate if you are eligible for preventive measures. The results of your investigation may turn out to be important for others in your family as well.

We are currently evaluating two different methods for disclosure of information within families. We do this in the form of a clinical trial called the DIRECT study. The study is coordinated from Umeå University in collaboration with Swedish cancer genetics clinics.

The purpose of the study is to evaluate if an additional service to standard care, namely sending information letters directly to eligible relatives, will affect how many relatives will then contact us for further support. We are also interested in how you as a patient experience this new practice.

How is the study conducted?

To participate in the study, you need to fulfill a number of criteria. If you fulfill the criteria and sign the consent form (on page 3), you will be randomized to one of the following two groups:

1. In the first group (control) you will be given standard care at the clinic. Upon receiving the results from your cancer genetic investigation, you will be encouraged to inform your relatives should the result turn out to be important also for them. As a help you may be offered written information in the form of a family letter.

2. In the second group (intervention) you will be offered the same standard care as those in group one, with the addition of healthcare offering to send direct, tailored personal letters to your relatives who may be concerned. You are still encouraged to inform those who you feel comfortable to discuss risk with.

Those participating in the study will be given two rounds of questionnaires delivered to you. The questionnaire will take about 10-15 minutes to complete, and participants are expected to allocate time for this. A few participants may also be asked to participate in a telephone interview.

Possible consequences of participating in the study

To participate in a cancer genetic investigation and learning about a hereditary cancer risk in the family is experienced by many as a necessary, but also sometimes stressful situation. The responsibility of informing at-risk relatives can be perceived as problematic and burdensome, but this discomfort would arise regardless of whether you participate in this study or not.

There are no known medical risks associated with participating in this study. Answering questionnaires and interview questions about family relationships and communication of disease risks can be emotional, if you find it difficult to think about your genetic investigation. However, there are several studies that show that knowledge of one's hereditary cancer risk, linked to receiving a follow-up program, reduces worry and anxiety compared to just suspecting a risk and not knowing what the actual risk is in the family

What happens to my personal data?

The project will collect and register information about you. The data is processed so that unauthorized persons cannot access it. When analyzing and reporting results, the material will be anonymous, and no individual person can be identified. Umeå University is responsible for your personal data. As an authority, Umeå University has an obligation to register and archive public documents. We therefore process your data to comply with applicable legislation and the legal basis for handling the data is public interest. Collected materials including consent documents are saved for 15 years.

According to the EU's data protection regulation, you have the right to access the information about you that is handled in the study free of charge, and if necessary to have any errors corrected. You can also request that information about you be deleted and that the processing of your personal data be restricted. However, the right to erasure and to limit the processing of personal data does not apply when the data is necessary for the current research.

If you want to access the data, you must contact the principal investigator responsible for the study (see contact details at the end of this letter). Umeå University's data protection officer can be reached by email: pulo@umu.se. If you are dissatisfied with how your personal data is processed, you have the right to submit a complaint to the Swedish Data Protection Agency (imy.se), which is the supervising authority.

How can I obtain the results from this study?

Results from this study will only be reported on group level. No single individual will be identifiable in either text or statistical graphs. The study results will be presented in scientific journals, at medical conferences and summarized in popular science formats, e.g., in public talks, posters or in digital formats.

Insurance and reimbursement

The national patient insurance is applicable as for any other care or treatment given within healthcare in Sweden. You will not receive any compensation for participation in this trial.

Participation is voluntary

If you chose to participate in the study, you may drop out at any time. If you chose to withdraw your consent you do not need to motivate your choice, and it will not affect any care or treatment in the future. If you want to withdraw from this study, please contact the Principal Investigator (contact details below).

Contact information to study staff

Lund: first.lastname@xxx.xx
Göteborg: first.lastname@xxx.xx
Stockholm: first.lastname@xxx.xx
Umeå: first.lastname@xxx.xx

Principal investigator (PI) for the DIRECT *study;*
Anna Rosén, MD, PhD and Specialist in Genetics,
Department of Radiation Sciences, Umeå University.
Telephone number: XXX-XXXX. Email: directstudien.rct@umu.se

Confirmation by participant: Consent to be included in the study

I have been given oral and written information about the DIRECT study and I have had the opportunity to ask questions. I will keep one copy of this written information for myself.

I consent to the handling of personal information about myself and my at-risk relatives according to the description in the patient information leaflet. I consent to being randomized to either group 1 (control) and be offered current standard practice or group 2 (intervention) where I will be offered a complementary offer in addition to current standard practice.

Personal number:	
City:	Signature:
Date (year, month, day)<br>_ _ _ _ / _ _ / _ _	Full name:

>>> Keep one copy of this written information (the white copy) and return the other (the blue copy) with your signature and date to the clinic by postal service, using the attached return envelope.

Contact details to clinics participating in the DIRECT study:

Norrlands universitetssjukhus
Att. First Last name
”DIRECT-studien”
Cancergenetisk Mottagning
Regionalt Cancercentrum Norr
Byggnad 5B, plan 0,
901 85 UMEÅ

Skånes universitetssjukhus
Att: First Last name
”DIRECT-studien”
Labmedicin
Klinisk Genetik
Akutgatan 8
221 85 Lund

Karolinska universitetssjukhuset Solna
Att: First Last name
”DIRECT-studien”
Mottagningen Klinisk Genetik
Karolinska vägen 37A, QB:84
171 76 Stockholm

Sahlgrenska universitetssjukhuset
Att: First Last name
”DIRECT-studien”
Klinisk genetik och genomik
41345 Göteborg
