# Supplementary material for: Direct letters to relatives at risk of hereditary cancer—study protocol for a multi-center randomized controlled trial of healthcare-assisted versus family-mediated risk disclosure at Swedish cancer genetics clinics (DIRECT-study)
Source: Trials. 2023 Dec 17;24:810. doi: 10.1186/s13063-023-07829-5 (PMC10726564; doi:10.1186/s13063-023-07829-5)
Supplement: Supplementary file 2 — Additional file 2. Case report forms. [file 13063_2023_7829_MOESM2_ESM.pdf]

**N.B.** The form is to be submitted to the study office after completed patient appointment.

# CRF 1 - Research subject data

Study site ◯ **Göteborg** ◯ **Linköping** ◯ **Lund** ◯ **Stockholm** ◯ **Umeå** ◯ **Uppsala** ◯ ________

## A. Personal data

| | | |
|---|---|---|
| Family number | Local identification number: | Cancer diagnosis or relapse in the last year<br>Yes ◯<br>No ◯ |
| Name | First name/ Last name ________ ________ | |
| Social security no. | YYYYMMDD-XXXX ☐☐☐☐ ☐☐ ☐☐ – ☐☐☐☐ | Patient prefers<br>Paper survey ◯<br>(record address)<br>E-survey online ◯<br>(record e-mail-address) |
| Contact info. | Address / e-mail | |

## B. Consent

| | |
|---|---|
| Date | ☐☐☐☐ ☐☐ ☐☐ |

## C. Inclusion criteria

| | |
|---|---|
| a) Referred to or contacted oncogenetic unit | **Yes** ◯ **No** ◯ |
| b) Returned signed consent form | **Yes** ◯ **No** ◯ |
| c) Is over 18 years of age | **Yes** ◯ **No** ◯ |
| d) Belongs to family with increased risk (**to D**) | **Yes** ◯ **No** ◯ |
| e) Has at least one at-risk relative to inform | **Yes** ◯ **No** ◯ |

## D. Family diagnosis

| | |
|---|---|
| 1. Familial breast cancer *(risk >20%)* | **Yes** ◯ |
| 2. Familial colorectal cancer *(>1 coloskopy)* | **Yes** ◯ |
| 3. Pathogenic variant in *BRCA1,BRCA2, PALB2* | **Yes** ◯ |
| 4. Lynch syndrome *(MLH1, MSH2, MSH6, PMS2, EPCAM)* | **Yes** ◯ |

◯ **Genetic screening** ◯ **Predictive testing**

## E. Randomization *(if patient fulfills all criteria in C)*

| | | |
|---|---|---|
| ◯ New patient | ◯ Previously randomized | DIRECT-participant number: ☐☐☐☐ – ☐☐ |

☐ Control ☐ Intervention

## F. Cancer genetic results discussed with patient

| | |
|---|---|
| Clinical relevance for at-risk relatives discussed with patient. (T=0). | Staff in charge of appointment: ________ |
| **Note:** Also fill in the CRF2-form with listing of p at-risk relatives. | Comment / note: |

**Date (T=0)**

☐☐☐☐ ☐☐ ☐☐

Date (YY/MM/DD)

## G. Transfer to research database *(for study staff in Umeå)*

| | | |
|---|---|---|
| Date of registration (YYYY/MM/DD)<br>☐☐☐☐ ☐☐ ☐☐<br>Signature | Other notes: | Adress to the study office:<br>**Norrlands universitetssjukhus**<br>**Regionalt cancercentrum Norr**<br>**Cancergenetisk mottagning**<br>**"DIRECT-studien" / Carin Nylander**<br>**901 85 UMEÅ** |

**Questions?** Please contact the national study coordinator by phone **076-69 60 648** or by email **directstudien.rct@umu.se**.

**N.B.** The form should be stored together with patient records in local clinical archive.

# CRF 2 - Family follow-up data

## A. Patient under investigation

| | | |
|---|---|---|
| Family number | Local identification number: | Assigned to study group<br>☐ Control<br>☐ Intervention |
| Study-specific family number | DIRECT-study participant number: ☐☐☐☐ - ☐☐ | |
| Name | First / Last name ______ ______ | Signature: |
| Social security no. | YYYY/MM/DD-XXXX ☐☐☐☐☐☐☐☐–☐☐☐☐ | |

**Total number** of at-risk relatives ☐☐

| | | | | |
|---|---|---|---|---|
| Sex | ☐☐ | Women | ☐☐ | Men |
| Relation | ☐☐ | 1st degree | ☐☐ | Other |

## B. At-risk relatives *(complete the list for all study participants)*

**1** ○ Sister ○ Brother ○ Mother ○ Father ○ ______

| | | |
|---|---|---|
| First- & Last name ______ | Is the at-risk relative already aware of the genetic investigation? | Yes ○ No ○ Unsure ○ |
| Street Address ______ | Has contaced oncogenetic clinic? Date | Yes ○ No ○ |
| Zip code ☐☐☐☐☐ City ______ | Address details sufficient to send DIRECT-letter? | Yes ○ No ○ |
| Date of birth (if known) + additional notes ☐☐☐☐☐☐☐☐ | Proband consented to contact the at-risk relative by letter? | Yes ○ No ○ |
| | DIRECT-letter sent to relative Date | Yes ○ No ○ |
| | Letter retrieved from postal office Date | Yes ○ No ○ |

**2** ○ Sister ○ Brother ○ Mother ○ Father ○ ______

| | | |
|---|---|---|
| First- & Last name ______ | Is the at-risk relative already aware of the genetic investigation? | Yes ○ No ○ Unsure ○ |
| Street Address ______ | Has contaced oncogenetic clinic? Date | Yes ○ No ○ |
| Zip code ☐☐☐☐☐ City ______ | Address details sufficient to send DIRECT-letter? | Yes ○ No ○ |
| ☐☐☐☐☐☐☐☐ | Proband consented to contact the at-risk relative by letter? | Yes ○ No ○ |
| | DIRECT-letter sent to relative Date | Yes ○ No ○ |
| | Letter retrieved from postal office Date | Yes ○ No ○ |

**>>>** Additional listing space on the next page

## 3

○ Sister ○ Brother ○ Mother ○ Father ○ ___________

| | | |
|---|---|---|
| First- & Last name ____________ | Is the at-risk relative already aware of the genetic investigation? | Yes ○ No ○ Unsure ○ |
| Street Address ____________ | Has contaced oncogenetic clinic? Date | Yes ○ No ○ |
| Zip code ☐☐☐ ☐☐ City ____________ | Address details sufficient to send DIRECT-letter? | Yes ○ No ○ |
| ☐☐☐☐ ☐☐ ☐☐ | Proband consented to contact the at-risk relative by letter? | Yes ○ No ○ |
| | DIRECT-letter sent to relative Date | Yes ○ No ○ |
| | Letter retrieved from postal office Date | Yes ○ No ○ |

## 4

○ Sister ○ Brother ○ Mother ○ Father ○ ___________

| | | |
|---|---|---|
| First- & Last name ____________ | Is the at-risk relative already aware of the genetic investigation? | Yes ○ No ○ Unsure ○ |
| Street Address ____________ | Has contaced oncogenetic clinic? Date | Yes ○ No ○ |
| Zip code ☐☐☐ ☐☐ City ____________ | Address details sufficient to send DIRECT-letter? | Yes ○ No ○ |
| Date of birth (if known) + additional notes ☐☐☐☐ ☐☐ ☐☐ | Proband consented to contact the at-risk relative by letter? | Yes ○ No ○ |
| | DIRECT-letter sent to relative Date | Yes ○ No ○ |
| | Letter retrieved from postal office Date | Yes ○ No ○ |

## 5

○ Sister ○ Brother ○ Mother ○ Father ○ ___________

| | | |
|---|---|---|
| First- & Last name ____________ | Is the at-risk relative already aware of the genetic investigation? | Yes ○ No ○ Unsure ○ |
| Street Address ____________ | Has contaced oncogenetic clinic? Date | Yes ○ No ○ |
| Zip code ☐☐☐ ☐☐ City ____________ | Address details sufficient to send DIRECT-letter? | Yes ○ No ○ |
| Date of birth (if known) + additional notes ☐☐☐☐ ☐☐ ☐☐ | Proband consented to contact the at-risk relative by letter? | Yes ○ No ○ |
| | DIRECT-letter sent to relative Date | Yes ○ No ○ |
| | Letter retrieved from postal office Date | Yes ○ No ○ |

Comment to the study secratariat:

**Questions?** Please contact the national study coordinator by phone **076-69 60 648** or by email **directstudien.rct@umu.se**.

**N.B.** The form is to be submittet to the study office after completion.

# CRF 3 - Outcome data

Study site ◯ **Göteborg** ◯ **Linköping** ◯ **Lund** ◯ **Stockholm** ◯ **Umeå** ◯ **Uppsala** ◯ ________

## A. Patient who started the investigation

| | | |
|---|---|---|
| Family number | Local unit/clinic number: | Assigned to study group<br>☐ Control<br>☐ Intervention |
| Study-specific family number | DIRECT-study participant number: ☐☐☐☐ - ☐☐ | |
| Name | First / Last Name ________ ________ | Type of investigation<br>☐ Gen.screen.<br>☐ Pred.testing |
| Social security no. | Date YYYY/MM/DD-XXXX ☐☐☐☐ ☐☐ ☐☐ – ☐☐☐☐ | |

## B. Evaluation of results

| | |
|---|---|
| Date when registers were checked<br>Relatives were followed-up (YYYY/MM/DD)<br>☐☐☐☐ ☐☐ ☐☐ | (First / Last name) ________ ________<br>Comments /notes: |

## C. Summary of main outcome after 12 months

| C. Summary of main outcome after 12 months | TOTAL | Women / Men | 1st degree / Other |
|---|---|---|---|
| I: Total number of relatives who could benefit from being informed | ☐☐ | ☐☐ / ☐☐ | ☐☐ / ☐☐ |
| II: Number of relatives who could not be identified / address unknown | ☐☐ | | |
| III: Number of relatives who contacted a cancer genetics clinic within 1 year | ☐☐ | ☐☐ / ☐☐ | ☐☐ / ☐☐ |

## D. Intervention - secondary outcomes

| D. Intervention - secondary outcomes | |
|---|---|
| I: Relatives the proband consented to contact by DIRECT-letter (number) | ☐☐ |
| II: Relatives who had already contacted a clinic before letter (number) | ☐☐ |
| III: Number of registered letters which were actually sent | ☐☐ |
| IX: Number of registered letters which were retrieved from the post office | ☐☐ |

---

## G. Transfer to research databas

| | | |
|---|---|---|
| Day of registration (YYYY/MM/DD)<br>☐☐☐☐ ☐☐ ☐☐<br>Signature: | Comments /notes for study administrator: | **Norrlands universitetssjukhus**<br>**Regionalt cancercentrum norr**<br>**Att. Carin Nylander**<br>**"DIRECT-studien"**<br>**Byggnad 5B, plan 0,**<br>**901 85 UMEÅ** |

**Questions?** Please contact the national study coordinator by phone **076-69 60 648** or by email **directstudien.rct@umu.se**.
