# Supplementary material for: Direct letters to relatives at risk of hereditary cancer—study protocol for a multi-center randomized controlled trial of healthcare-assisted versus family-mediated risk disclosure at Swedish cancer genetics clinics (DIRECT-study)
Source: Trials. 2023 Dec 17;24:810. doi: 10.1186/s13063-023-07829-5 (PMC10726564; doi:10.1186/s13063-023-07829-5)
Supplement: Supplementary file 3 — Additional file 3. Progress criteria and study site audit. [file 13063_2023_7829_MOESM3_ESM.pdf]

Table S1. Progress criteria and study site audit measures

	Question	Pilot outcome	Progression criteria
Clinical uncertainties	Is the direct letter an accepted mode of contact with relatives by participants?	Proportion of previously unnotified relatives that the participant accepts to be contacted by letter	Go: 40-100%<br>Amend: 20-40%<br>Alert: <20%
	Is the psychological reaction to listing details of relatives acceptable during the counselling session?	Any report of severe adverse effects from the listing of relatives among the 20 individuals in the internal pilot.	Go: No reports<br>Amend: 1-2 reports<br>Alert: >2 reports
	How will at-risk relatives react when they pick up a direct letter with information about hereditary cancer risk?	Any report of severe adverse effects from at-risk relatives as response to receiving letter.	Go: No reports<br>Amend: 1-2 reports<br>Alert: >2 reports
Procedural uncertainties	How many eligible patients are invited to join the trial per study site and time?	Number of invited patients per center and month.	Go: >1 patient.<br>Amend: 0-1 patient.<br>(If 0 included the study site may be discontinued.)
	How many eligible patients were invited to join the trial per each study site´s recruitment basis?	Number of invited patients during one month / number of clinical admissions of eligible patients the same month.	Go: >40%<br>Amend: 10-40%<br>Alert: <10%
	Inclusion: How many patients did NOT accept invitation to participate in the study?	Number of patients who decline or do not return their consent form within 4 weeks / number of patients invited.	Go: <30%<br>Amend: 30-50%<br>Alert: >50%
Procedural uncertainties (cont.)	How many patients return their Consent Forms to the clinics?	Number of Consent forms returned at IPP (internal pilot point) per invited patients 4 weeks before IPP and studysite.	Go: >40%<br>Amend: 10-40%<br>Alert: <10%

	Is the added time required to administer intervention acceptable in the clinical setting?	Estimated working time per at-risk relative by research nurses and/or trial physicians.	Go: <1 hours<br>Amend: 1min-3h<br>Alert: >3h
	Is it possible to retrieve the data for the final outcome from local patient registries within an acceptable time?	Reported estimated time to fill in the CRF3 per participant.	Go: <2 hour<br>Amend: 2-6 hour<br>Alert: >6 hours
Methodological uncertainties	Does the HCP treat control and intervention group according to study protocol.	Observational data from patient visits reporting deviation from protocol.	Go: No reports<br>Amend: 0-1 reports<br>Alert: >1 reports
	How will the participants in the control arm react when they are NOT offered the service of direct letters to relatives?	Drop-out rates when finding out one has been randomized to the control arm.	Go: 0 drop-outs<br>Amend: 1-2 drop-out<br>Alert: >2 drop-outs
