# Supplementary material for: Direct letters to relatives at risk of hereditary cancer—study protocol for a multi-center randomized controlled trial of healthcare-assisted versus family-mediated risk disclosure at Swedish cancer genetics clinics (DIRECT-study)
Source: Trials. 2023 Dec 17;24:810. doi: 10.1186/s13063-023-07829-5 (PMC10726564; doi:10.1186/s13063-023-07829-5)
Supplement: Supplementary file 4 — Additional file 4. Letter templates. [file 13063_2023_7829_MOESM4_ESM.pdf]

TEMPLATES FOR DIRECT LETTERS

8 diagnose-specific templates for letters addressing at-risk relatives in the DIRECT-study

1. Familial Breast Cancer
2. Familial Colorectal Cancer
3. Pathogenic Variant in BRCA1 (female recipient)
4. Pathogenic Variant in PALB2 (female recipient)
5. Pathogenic Variant in BRCA1 (male recipient)
6. Pathogenic Variant in PALB2 (male recipient)
7. Pathogenic Variant in BRCA2 (male and female recipients)
8. Pathogenic Variant in MLH1, MSH2, MSH6, PMS2 (male and female recipients)

PI: Anna Rosén

The DIRECT-study, Umeå university

Familial Breast Cancer

Instruction: This letter is sent to

- *Relatives who according to standard clinical evaluation should be offered some form of surveillance programme AND who are currently within the recommended age interval for surveillance, i.e is recommended a mammography exam within 12 months.*

Hello,

You are receiving this letter from a healthcare prefessional who wants to inform you of a medical investigation which concerns your family/kinship. This information is not urgent. We have sent you this letter by certified mail to ensure the letter is delivered and reaches the right person.

A relative of yours (XX- optional to include proband's name here) has undergone an investigation at our clinic and you may thus already be aware of the information below.

We have investigated if women in your family may have an increased risk of breast cancer and whether they should be offered a surveillance programme as a prevention strategy.

According to our assessment there may be an increased risk of breast cancer in your family, but we have not identified a specific gene variant as an underlying cause of cancer. We therefore offer regular exams to all individuals in your family who have been diagnosed with cancer, as well as their sisters, daughters and mothers.

The results of your relative's investigation concerns you since we want to offer you annual breast exams, called mammography, starting from the age of **XX** years – instead of every annual year which is the common interval offered to all women over 40 years in Sweden.

For more information you are welcome to contact us using the details below. We can offer more information about the investigation and how you may obtain a referral for an exam. Details of all cancer genetic clinics in Sweden are available online at www.1177.se/cancergenetik.

Name (Nurse, Physician)
Tel: xxxx (to the nurse/physician/local clinic)
Webpage on national health platform
Cancer genetic clinic in XXX

Familial Colorectal Cancer

Instruction: This letter is sent to:

- *Relatives who according to standard clinical evaluation guidelines should be offered some form of surveillance programme AND who are currently within the recommended age interval for surveillance.*

Hello,
You are receiving this letter from a healthcare professional who wants to inform you of a medical investigation which concerns your family/kinship. This information is not urgent. We have sent you this letter by certified mail to ensure the letter is delivered and reaches the right person.

A relative of yours (XX- optional to include probands name here) has undergone an investigation at our clinic and you may thus already be aware of the information below.

We have investigated if there may be an increased risk of colorectal cancer in your family and whether individuals in your family should be offered a surveillance programme as a prevention strategy.

According to our assessment there may be an increased risk of colorectal cancer in your family, but we have not identified a specific gene variant as an underlying cause of cancer. We therefore offer regular exams to all individuals in your family who have been diagnosed with colorectal cancer, as well as their siblings, children and parents.

This investigation concerns you since we want to offer you regular bowel exams, called colonoscopies, every fifth year from the age of **XX years**. A colonoscopy involves examining the colon using a soft, flexible tube with a camera. This enables early detection, and if necessary, removal of changes which may lead to cancer.

For more information you are welcome to contact us using the details below. We can offer more information about the investigation and how you may obtain a referral for an exam. Details of all cancer genetic clinics in Sweden are available online at www.1177.se/cancergenetik.

Name (Nurse, Physician)
Tel: xxxx (to the nurse/physician/local clinic)
Webpage on national health platform
Cancer genetic clinic in XXX

Pathogenic Variant in BRCA1 (female recipient)

Instruction: This letter is sent to

- *Those who according to standard clinical evaluation guidelines should be offered genetic testing at this stage in the family cascade (more individuals in the same family may be included futher on, but at a later stage).*

Hello,
You are receiving this letter from a healthcare prefessional who wants to inform you of a medical investigation which concerns your family/kinship. This information is not urgent. We have sent you this letter by certified mail to ensure the letter is delivered and reaches the right person.

A relative of yours (XX- optional to include probands name here) has undergone an investigation at our clinic and you may thus already be aware of the information below.

In your family we have identified a pathogenic gene variant which is related to an increased risk of developing certain types of cancers (breast and ovarian cancer). It is possible to find out if you have inherited the variant or not by taking a blood sample. You are welcome to contact us for more information on how an investigation is conducted and the implications for yourself and your family.

If it turns out you do carry the pathogenic gene variant we can offer you a preventive surveillance programme which include regular exams of the breasts and ovaries. We can also offer your adult children information and predictive testing.

If you do not carry the gene variant you have no increased risk of these types of cancers and you cannot pass the variant on to the next generation.

For more information you are welcome to contact us using the details below. The investigation is offered at a cancer genetics clinic in your region. Details of all cancer genetic clinics in Sweden are available online at www.1177.se/cancergenetik.

Name (Nurse, Physician)
Tel: xxxx (to the nurse/physician/local clinic)
Webpage on national health platform
Cancer genetic clinic in XXX

Pathogenic Variant in PALB2 (female recipient)

Instruction: This letter is sent to

- *Those who according to standard clinical evaluation guidelines should be offered genetic testing at this stage in the family cascade (more individuals in the same family may be included futher on, but at a later stage).*

Hello,
You are receiving this letter from a healthcare prefessional who wants to inform you of a medical investigation which concerns your family/kinship. This information is not urgent. We have sent you this letter by certified mail to ensure the letter is delivered and reaches the right person.

A relative of yours (XX- optional to include probands name here) has undergone an investigation at our clinic and you may thus already be aware of the information below.

In your family we have identified a pathogenic gene variant which is related to an increased risk of developing certain types of cancers (breast and ovarian cancer). It is possible to find out if you have inherited the variant or not by taking a blood sample. You are welcome to contact us for more information on how an investigation is conducted and the implications for yourself and your family.

If it turns out you do carry the pathogenic gene variant we can offer you a preventive surveillance programme which include regular exams of the breasts and ovaries. We can also offer your adult children information and predictive testing.

If you do not carry the gene variant you have no increased risk of these types of cancers and you cannot pass the variant on to the next generation.

For more information you are welcome to contact us using the details below. The investigation is offered at a cancer genetics clinic in your region. Details of all cancer genetic clinics in Sweden are available online at www.1177.se/cancergenetik.

Name (Nurse, Physician)
Tel: xxxx (to the nurse/physician/local clinic)
Webpage on national health platform
Cancer genetic clinic in XXX

Pathogenic Variant in BRCA1 (male recipient)

Instruction: This letter is sent to

- *Those who according to standard clinical evaluation guidelines should be offered genetic testing at this stage in the family cascade (more individuals in the same family may be included futher on, but at a later stage).*

Hello,
You are receiving this letter from a healthcare prefessional who wants to inform you of a medical investigation which concerns your family/kinship. This information is not urgent. We have sent you this letter by certified mail to ensure the letter is delivered and reaches the right person.

A relative of yours (XX- optional to include probands name here) has undergone an investigation at our clinic and you may thus already be aware of the information below.

In your family we have identified a pathogenic gene variant which is related to an increased risk of developing certain types of cancers (breast and ovarian cancer). It is possible to find out if you have inherited the variant or not by taking a blood sample. You are welcome to contact us for more information on how an investigation is conducted and the implications for yourself and your family.

Men carry this variant as often as women. The variant can be passed on to the next generation, which is why it is important also for men to clarify if they are carriers of this variant .

If it turns out you do carry the pathogenic gene variant we can offer your adult children to take a blood sample to find out if they have inherited the variant. If a man is a carrier he can pass on the variant to his children and this information may be important for them as well. If a woman is a carrier we are able to offer her a preventive surveillance programme.

If you do not carry the gene variant you cannot pass on the variant to the next generation.

For more information you are welcome to contact us using the details below. The investigation is offered at a cancer genetics clinic in your region. Details of all cancer genetic clinics in Sweden are available online at www.1177.se/cancergenetik.

Name (Nurse, Physician)
Tel: xxxx (to the nurse/physician/local clinic)
Webpage on national health platform
Cancer genetic clinic in XXX

Pathogenic Variant in PALB2 (male recipient)

Instruction: This letter is sent to

- *Those who according to standard clinical evaluation guidelines should be offered genetic testing at this stage in the family cascade (more individuals in the same family may be included futher on, but at a later stage).*

Hello,
You are receiving this letter from a healthcare prefessional who wants to inform you of a medical investigation which concerns your family/kinship. This information is not urgent. We have sent you this letter by certified mail to ensure the letter is delivered and reaches the right person.

A relative of yours (XX- optional to include probands name here) has undergone an investigation at our clinic and you may thus already be aware of the information below.

In your family we have identified a pathogenic gene variant which is related to an increased risk of developing certain types of cancers (breast and ovarian cancer). It is possible to find out if you have inherited the variant or not by taking a blood sample. You are welcome to contact us for more information on how an investigation is conducted and the implications for yourself and your family.

Men carry this variant as often as women. The variant can be passed on to the next generation, which is why it is important also for men to clarify if they are carriers of this variant .

If it turns out you do carry the pathogenic gene variant we can offer your adult children to take a blood sample to find out if they have inherited the variant. If a man is a carrier he can pass on the variant to his children and this information may be important for them as well. If a woman is a carrier we are able to offer her a preventive surveillance programme.

If you do not carry the gene variant you cannot pass on the variant to the next generation.

For more information you are welcome to contact us using the details below. The investigation is offered at a cancer genetics clinic in your region. Details of all cancer genetic clinics in Sweden are available online at www.1177.se/cancergenetik.

Name (Nurse, Physician)
Tel: xxxx (to the nurse/physician/local clinic)
Webpage on national health platform
Cancer genetic clinic in XXX

Pathogenic Variant in *BRCA2* (male and female recipients)

Instruction: This letter is sent to

- *Those who according to standard clinical evaluation guidelines should be offered genetic testing at this stage in the family cascade (more individuals in the same family may be included futher on, but at a later stage).*

Hello,
You are receiving this letter from a healthcare prefessional who wants to inform you of a medical investigation which concerns your family/kinship. This information is not urgent. We have sent you this letter by certified mail to ensure the letter is delivered and reaches the right person.

A relative of yours (XX- optional to include probands name here) has undergone an investigation at our clinic and you may thus already be aware of the information below.

In your family we have identified a pathogenic gene variant which is related to an increased risk of developing certain types of cancers (breast, ovarian and prostate cancer). It is possible to find out if you have inherited the variant or not by taking a blood sample. You are welcome to contact us for more information on how an investigation is conducted and what this means for yourself and your family.

If it turns out you do carry the pathogenic gene variant we can offer you a surveillance programme. The programme for women includes regular exams of breasts and ovaries. Men are offered regular prostate exams. If your carry the variant we can also offer your adult children information and predictive testing.

If you do not carry the gene variant you have no increased risk of these types of cancers and you cannot pass such a variant on to the next generation.

For more information you are welcome to contact us using the details below. The investigation is offered at a cancer genetics clinic in your region. Details of all cancer genetic clinics in Sweden are available online at www.1177.se/cancergenetik.

Name (Nurse, Physician)
Tel: xxxx (to the nurse/physician/local clinic)
Webpage on national health platform
Cancer genetic clinic in XXX

Pathogenic Variant i MLH1, MSH2, MSH6 and PMS2 (male and female recipients)

Instruction: This letter is sent to

- *Those who according to standard clinical evaluation guidelines should be offered genetic testing at this stage in the family cascade (more individuals in the same family may be included futher on, but at a later stage).*

Hello,
You are receiving this letter from a healthcare prefessional who wants to inform you of a medical investigation which concerns your family/kinship. This information is not urgent. We have sent you this letter by certified mail to ensure the letter is delivered and reaches the right person.

A relative of yours (XX- optional to include probands name here) has undergone an investigation at our clinic and you may thus already be aware of the information below.

In your family we have identified a pathogenic gene variant which is related to an increased risk of certain types of cancers (colon, uterine and ovarian cancer). It is possible to find out if you have inherited the variant or not by taking a blood sample. You are welcome to contact us for more information on how an investigation is conducted and the implications for yourself and your family.

If it turns out you do carry the pathogenic variant we can offer you a preventive surveillance programme. You would then be eligible to undergo regular bowel exams, also called colonoscopy. A colonoscopy involves examining the colon using a soft, flexible tube with a camera. This enables early detection, and if necessary, removal of changes which may lead to cancer. Women are in addition offered to attend gynecological health exams. If your carry the variant we can also offer your adult offspring information and predictive testing.

If you do not carry the pathogenic variant you have no increased risk of these types of cancers and you cannot pass such a variant on to the next generation.

For more information you are welcome to contact us using the details below. The investigation is offered at a cancer genetics clinic in your region. Details of all cancer genetic clinics in Sweden are available online at www.1177.se/cancergenetik.

Name (Nurse, Physician)
Tel: xxxx (to the nurse/physician/local clinic)
Webpage on national health platform
Cancer genetic clinic in XXX
