# Supplementary material for: Direct letters to relatives at risk of hereditary cancer—study protocol for a multi-center randomized controlled trial of healthcare-assisted versus family-mediated risk disclosure at Swedish cancer genetics clinics (DIRECT-study)
Source: Trials. 2023 Dec 17;24:810. doi: 10.1186/s13063-023-07829-5 (PMC10726564; doi:10.1186/s13063-023-07829-5)
Supplement: Supplementary file 5 — Additional file 5. Questionnaires. [file 13063_2023_7829_MOESM5_ESM.pdf]

Questionnaires administrated to probands at T0 (0 months) and T2 (6 months)

Important note:
The translation of the questionnaires is made by the authors.

Translation of text starts below. The original instruments are not translated here.

Questionnaire administrated to probands at T0 (0 months)

Page 1
DIRECT study questionnaire T0

How did you experience participating in a cancergenetic investigation?
Questionnaire 1 of 2

Hello!

You have previously agreed to participate in a research study called the DIRECT study, and we are now asking you to answer this questionnaire. It takes about 15 minutes depending on how much free text you write.

We are conducting the study to increase knowledge about individuals' experience of learning about a hereditary increased risk of cancer. Your thoughts and opinions are important so that we can evaluate the way we work and develop well-functioning routines that give our patients the best possible support.

Your answers will be handled anonymously and safely. Only authorized researchers will have access to the data. Analyses and reporting are done at group level.

All personal data is handled in accordance with the provisions of the Data Protection Regulation (GDPR) and the Personal Data Act (PuL). You have the right to access, or correct, the information about you once a year by contacting the person in charge of personal data at Umeå University. Contact details are on the back of the form.

When you have completed the questions, post the form in the attached return postage envelope. No stamp is needed. If you have misplaced the envelope, you can find the address on the back.

Through your answer, you contribute to developing routines at Swedish cancer genetic clinics.

Page 2 and 3
Swedish version of RAND-36. Distributed by Registercentrum Sydost (RCSO), version 2013-05-21

Page 4 and 5
Swedish version of STAI-S and STAI-T. Free Swedish version adopt from
Spielberger, C.D., Gorsuch, R.L., & Lushene, R.E. (1970). The State-Trait Anxiety Inventory: Testmanual. Palo Alto, CA: Consulting Psychologist Press

Page 6 and 7

Swedish version of Cancer worry scale. Adapted from Lerman C, 1994, Douma KH, 2010, Lammens CRM, 2010 and translated to Swedish.

Page 8 and 9
Your experiences of the cancer genetics clinic
Below you find a couple of open questions about your contact with the cancer genetics clinic. We want you to honestly share your thoughts and experiences of participating in a family investigation at the clinic.

GC01. How did you feel that you were treated by the staff at the clinic?
<free text>
GC02. How did you percieve the information you received?
<free text>
GC03. Do you have any suggestions on how the work at our clinic could be improved?
<free text>
GC04. If you want to add something that we haven't asked about, please write this below.
<free text>
GC05. If something is unclear in the questionnaire, for example if we notice that we have asked an unclear question, may we contact you for clarification?
Yes/No (add phonenumber)

a. Some brief information about you

Finally, we would like you to answer a short question about yourself

b. What is your highest level of education?

- ☐ Less than 9 years of schooling
- ☐ Completed primary school or the equivalent of 9 years of schooling
- ☐ Completed upper secondary school or the equivalent of 12 years of schooling
- ☐ Education at least 1 year beyond secondary school
- ☐ Degree from university or college

Page 10
Thank you for taking the time to share your experiences.

You are welcome to contact us if you have any questions or concerns about the study.

the DIRECT study
Direct information to at-risk relatives – A randomized controlled trial

Study phone: 076-69 60 648
E-mail: directstudien.rct@umu.se
Responsible researcher: Anna Rosén
Department of radiation sciences
Umeå university
SE-901 87 Umeå

UMEÅ UNIVERSITET

Questionnaires administrated to probands at T2 (6 months)

Page 1
DIRECT study questionnaire T6

Hello!

You have previously agreed to participate in a research study called the DIRECT study, and we are now asking you to answer this questionnaire

We are conducting the study to increase knowledge about individuals' experience of learning about a hereditary increased risk of cancer. We have previously asked you to answer these questions, but to be able to study changes in well-being over time we ask you to answer the same questions once again.

It takes about 10 minutes depending on how much free text you write.

Your answers will be handled anonymously and safely. Only authorized researchers will have access to the data. Analyses and reporting are done at group level.

All personal data is handled in accordance with the provisions of the Data Protection Regulation (GDPR) and the Personal Data Act (PuL). You have the right to access, or correct, the information about you once a year by contacting the person in charge of personal data at Umeå University. Contact details are on the back of the form.

This is how you return the questionnaire
When you have have completed the questionnaire, post the form in the attached return postage envelope. No stamp is needed. If you have misplaced the envelope, the address is:

Norrlands universitetssjukhus
DIRECT-studien
Cancergenetisk mottaning
Regionalt cancercentrum Norr
Byggnad 5B, plan 0
90158 Umeå

Page 2 and 3
Swedish version of RAND-36. Distributed by Registercentrum Sydost (RCSO), version 2013-05-21

Page 4 and 5
Swedish version of STAI-S and STAI-T. Free Swedish version adopt from *Spielberger, C.D., Gorsuch, R.L., & Lushene, R.E. (1970). The State-Trait Anxiety Inventory: Testmanual. Palo Alto, CA: Consulting Psychologist Press*

Page 6 and 7
Swedish version of Cancer worry scale. Adapted from Lerman C, 1994, Douma KH, 2010, Lammens CRM, 2010 and translated to Swedish.

If you want to add something that we haven't asked about, please write this below.
<free text>

Page 8

Thank you for taking the time to share your experiences.

You are welcome to contact usif you have any questions or concerns about the study.

the DIRECT study
Direct information to at-risk relatives – A randomized controlled trial

Study phone: 076-69 60 648
E-mail: directstudien.rct@umu.se
Responsible researcher: Anna Rosén
Department of radiation sciences
Umeå university
SE-901 87 Umeå

UMEÅ UNIVERSITET

Hur har du upplevt att delta i en cancergenetisk utredning?

Enkät 1 av 2

Hej!

Du har tidigare samtyckt till att delta i en forskningsstudie som kallas DIRECT-studien, och vi ber dig besvara detta frågeformulär. Det tar cirka 15 minuter beroende på hur mycket fri text du skriver.

Vi genomför studien för att öka kunskapen om individers upplevelse att få kännedom om en ärftlig förhöjd risk för cancer. Dina tankar och synpunkter är viktiga för att vi ska kunna utvärdera vår verksamhet och utveckla väl fungerande metoder som ger våra patienter bästa möjliga stöd.

Dina svar kommer att behandlas anonymt och säkert. Endast behöriga forskare kommer att ta del av datat. Analyser och rapportering görs på gruppnivå.

Alla personuppgifter hanteras enligt bestämmelser i dataskydds-förordningen (GDPR) och personuppgiftslagen (PuL). Du har rätt att få tillgång till, eller rätta, uppgifterna om dig en gång per år genom kontakt med personuppgiftsansvarig vid Umeå universitet. Kontaktuppgifter finns på baksidan av formuläret.

Returnera enkäten så här
När du fyllt i alla frågor, postar du formuläret i bifogat svarspostkuvert. Inget frimärke behövs. Om du tappat bort kuvertet så finns adress på baksidan.

Genom ditt svar bidrar du till att utveckla verksamheten vid svenska cancergenetiska mottagningar.

RAND-36 Hälsa och livskvalitet

RAND-36 handlar om din hälsa och funktion i vardagen. Välj det svarsalternativ som stämmer bäst för dig på varje fråga.

	Utmärkt	Mycket god	God	Någorlunda	Dålig
1. I allmänhet, skulle du säga att din hälsa är:	☐	☐	☐	☐	☐

	Mycket bättre	Något bättre	Ungefär densamma	Något sämre	Mycket sämre
2. **Jämfört med för ett år sedan,** hur skulle du bedöma din hälsa **nu**?	☐	☐	☐	☐	☐

Följande frågor handlar om aktiviteter du kan tänkas ägna dig åt en vanlig dag. **Begränsar din nuvarande hälsa dig** i dessa aktiviteter? Om ja, hur mycket?

	Ja, mycket begränsad	Ja, lite begränsad	Nej, inte alls begränsad
3. **Fysiskt ansträngande aktiviteter**, t.ex. löpning, lyfta tunga föremål, delta i ansträngande idrotter	☐	☐	☐
4. **Måttligt ansträngande aktiviteter**, t.ex. flytta ett bord, dammsuga, promenera eller cykla	☐	☐	☐
5. Lyfta eller bära matkassar	☐	☐	☐
6. Gå upp för **flera** trappor	☐	☐	☐
7. Gå upp för **en** trappa	☐	☐	☐
8. Böja dig eller gå ner på knä	☐	☐	☐
9. Gå **mer än ett par kilometer**	☐	☐	☐
10. Gå **flera kvarter** (flera hundra meter)	☐	☐	☐
11. Gå **ett kvarter** (hundra meter)	☐	☐	☐
12. Bada/duscha eller klä på dig	☐	☐	☐

Under de **senaste 4 veckorna**, har du haft något av följande problem med ditt arbete eller andra vanliga dagliga aktiviteter **på grund av din fysiska hälsa**?

	Ja	Nej
13. Dragit ner på **tiden** du ägnat åt arbete eller andra aktiviteter	☐	☐
14. **Fått mindre gjort** än du skulle vilja	☐	☐
15. Begränsats i **vissa** arbetsuppgifter eller andra aktiviteter	☐	☐
16. Haft **svårt** att utföra arbete eller andra aktiviteter (t.ex. det krävdes mer ansträngning)	☐	☐

Under de **senaste 4 veckorna**, har du haft något av följande problem med ditt arbete eller andra vanliga dagliga aktiviteter **på grund av känslomässiga problem** (t.ex. att du känt dig nere eller orolig)?

	Ja	Nej
17. Dragit ner på **tiden** du ägnat åt arbete eller andra aktiviteter	☐	☐
18. **Fått mindre gjort** än du skulle vilja	☐	☐
19. Utfört arbete eller andra aktiviteter mindre **noggrant** än vanligt	☐	☐

RAND Corporation, USA, äger upphovsrätt till originalet, som togs fram inom Medical Outcomes Study.
Registercentrum Sydost (RCSO) distribuerar översättningen av RAND-36, svensk version 2013-05-21.

	Inte alls	Lite grand	Måttligt	Ganska mycket	Extremt mycket
20. Under de **senaste 4 veckorna**, i vilken omfattning har din fysiska hälsa eller känslomässiga problem stört dina vanliga sociala aktiviteter med familj, släkt, vänner, grannar eller föreningar etc.?	☐	☐	☐	☐	☐

	Ingen	Mycket lätt	Lätt	Måttlig	Svår	Mycket svår
21. Hur mycket **fysisk** smärta har du haft under de **senaste 4 veckorna**?	☐	☐	☐	☐	☐	☐

	Inte alls	Lite grand	Måttligt	Ganska mycket	Extremt mycket
22. Under de **senaste 4 veckorna**, hur mycket har smärta stört ditt vanliga arbete (gäller både arbete utanför hemmet och hushållsarbete)?	☐	☐	☐	☐	☐

Följande frågor handlar om hur du känner dig och hur det har varit **under de senaste 4 veckorna**. Ange det svar som stämmer bäst med hur du känt dig.

Hur mycket av tiden under de **senaste 4 veckorna** ...	Hela tiden	Största delen av tiden	En stor del av tiden	En viss del av tiden	En liten del av tiden	Inget av tiden
23. Har du känt dig pigg?	☐	☐	☐	☐	☐	☐
24. Har du känt dig mycket nervös?	☐	☐	☐	☐	☐	☐
25. Har du känt dig så nere att ingenting kunnat muntra upp dig?	☐	☐	☐	☐	☐	☐
26. Har du känt dig lugn och harmonisk?	☐	☐	☐	☐	☐	☐
27. Har du känt dig energisk?	☐	☐	☐	☐	☐	☐
28. Har du känt dig dyster och ledsen?	☐	☐	☐	☐	☐	☐
29. Har du känt dig utsliten?	☐	☐	☐	☐	☐	☐
30. Har du känt dig lycklig?	☐	☐	☐	☐	☐	☐
31. Har du känt dig trött?	☐	☐	☐	☐	☐	☐

	Hela tiden	Största delen av tiden	En viss del av tiden	En liten del av tiden	Inget av tiden
32. Under de **senaste 4 veckorna**, hur mycket av tiden har din **fysiska hälsa eller känslomässiga problem** stört dina sociala aktiviteter (som att träffa vänner, släktingar etc.)?	☐	☐	☐	☐	☐

Hur väl stämmer följande påståenden in på dig?

	Stämmer helt	Stämmer ganska bra	Vet inte	Stämmer ganska dåligt	Stämmer inte alls
33. Jag verkar ha något lättare att bli sjuk än andra människor	☐	☐	☐	☐	☐
34. Jag är lika frisk som andra jag känner	☐	☐	☐	☐	☐
35. Jag tror att min hälsa kommer att försämras	☐	☐	☐	☐	☐
36. Min hälsa är utmärkt	☐	☐	☐	☐	☐

RAND Corporation, USA, äger upphovsrätt till originalet, som togs fram inom Medical Outcomes Study.
Registercentrum Sydost (RCSO) distribuerar översättningen av RAND-36, svensk version 2013-05-21.

Nr: _ _ _

STAI- S

Instruktioner: Här nedan följer några påståenden som beskriver hur man kan känna sig. Läs varje påstående noga och ringa sedan in den siffra som bäst beskriver hur du känner dig just nu, i detta ögonblick. Det finns inga "rätt" eller "fel". Använd inte för mycket tid för varje påstående utan svara utifrån det du tycker bäst beskriver dina nuvarande känslor.

Siffrorna har följande betydelse:

1 = Inte alls, 2 = Något, 3 = En hel del, 4 = Väldigt mycket.

1. Jag känner mig lugn	1	2	3	4
2. Jag känner mig trygg	1	2	3	4
3. Jag är spänd	1	2	3	4
4. Jag är ångestfylld	1	2	3	4
5. Jag känner mig väl till mods	1	2	3	4
6. Jag känner mig upprörd	1	2	3	4
7. Jag oroar mig över tänkbara olyckor	1	2	3	4
8. Jag känner mig utvilad	1	2	3	4
9. Jag känner mig ängslig	1	2	3	4
10. Jag känner välbefinnande	1	2	3	4
11. Jag känner mig självsäker	1	2	3	4
12. Jag känner mig nervös	1	2	3	4
13. Jag är uppjagad	1	2	3	4
14. Jag känner mig överkänslig	1	2	3	4
15. Jag är avslappnad	1	2	3	4
16. Jag känner mig belåten	1	2	3	4
17. Jag är orolig	1	2	3	4
18. Jag känner mig överspänd och "skakis"	1	2	3	4
19. Jag känner mig glad	1	2	3	4
20. Jag känner mig angenäm	1	2	3	4

STAI-T

Instruktioner: Här nedan följer några påståenden som beskriver hur man kan känna sig. Läs varje påstående noga och ringa sedan in den siffra som bäst beskriver hur du vanligen känner dig. Det finns inga "rätt" eller "fel". Använd inte för mycket tid för varje påstående utan svara det som du tycker bäst beskriver hur du vanligen känner dig.

Siffrorna har följande betydelse:

1 = Nästan aldrig, 2 = Ibland, 3 = Ofta, 4 = Nästan alltid.

1. Jag känner mig angenäm	1	2	3	4
2. Jag blir fort trött	1	2	3	4
3. Jag känner mig gråtfärdig	1	2	3	4
4. Jag önskar jag kunde vara lika lycklig som andra verkar vara	1	2	3	4
5. Jag går miste om saker för att jag inte kan bestämma mig fort nog	1	2	3	4
6. Jag känner mig utvilad	1	2	3	4
7. Jag är "lugn och samlad"	1	2	3	4
8. Jag känner att svårigheter växer sig så stora att jag inte klarar av dem	1	2	3	4
9. Jag oroar mig för mycket över saker som egentligen inte spelar någon roll	1	2	3	4
10. Jag är lycklig	1	2	3	4
11. Jag är benägen att ta saker och ting för allvarligt	1	2	3	4
12. Jag saknar självförtroende	1	2	3	4
13. Jag känner mig trygg	1	2	3	4
14. Jag försöker undvika att ta itu med svårigheter och kriser	1	2	3	4
15. Jag känner mig nedstämd	1	2	3	4
16. Jag är belåten	1	2	3	4
17. Någon oväsentlig tanke far genom mitt huvud och stör mig	1	2	3	4
18. Jag tar besvikelser så hårt att jag ej kan släppa dem ur mina tankar	1	2	3	4
19. Jag är en stabil person	1	2	3	4
20. Jag blir spänd och upprörd när jag tänker på mina aktuella bekymmer	1	2	3	4

Instrument för kvalitativ uppföljning av forskningspersoners upplevelser (STAI, 2 av 2 sidor).
Referens: *Spielberger, C.D., Gorsuch, R.L., & Lushene, R.E. (1970). The State-Trait Anxiety Inventory: Testmanual. Palo Alto, CA: Consulting Psychologist Press*

Cancer Worry Scale

Adapted from Lerman C, 1994, Douma KH, 2010, Lammens CRM, 2010 and translated to Swedish.

Nu följer ett antal frågor om dina tankar och känslor kring cancer. Frågorna gäller oavsett om du har cancer eller inte.

Q21

Hur ofta har du tänkt på risken att utveckla cancer (eller att utveckla cancer igen)?

- ☐ 1 Sällan eller aldrig
- ☐ 2 Ibland
- ☐ 3 Ofta
- ☐ 4 Nästan hela tiden

Q22

Har dessa tankar påverkat ditt humör?

- ☐ 1 Inte alls
- ☐ 2 Lite
- ☐ 3 En del
- ☐ 4 Mycket

Q23

Har dessa tankar påverkat din förmåga att utföra dagliga sysslor?

- ☐ 1 Inte alls
- ☐ 2 Lite
- ☐ 3 En del
- ☐ 4 Mycket

Q24

Hur orolig är du över risken att någon gång utveckla cancer (eller att utveckla cancer igen)?

- ☐ 1 Inte alls orolig
- ☐ 2 Inte särskilt orolig
- ☐ 3 Ganska orolig
- ☐ 4 Mycket orolig

Q25

Hur ofta oroar du dig över att utveckla cancer (eller att utveckla cancer igen)?

- ☐ 1 Sällan eller aldrig
- ☐ 2 Ibland
- ☐ 3 Ofta
- ☐ 4 Nästan hela tiden

Q26

Hur stort problem är denna oro?

- ☐ 1 Mycket litet
- ☐ 2 Ganska litet
- ☐ 3 Ganska stort
- ☐ 4 Mycket stort

Q27

Hur ofta oroar du dig för risken att dina familjemedlemmar kommer att utveckla cancer?

- ☐ 1 Sällan eller aldrig
- ☐ 2 Ibland
- ☐ 3 Ofta
- ☐ 4 Nästan hela tiden

Q28

Hur orolig är du över möjligheten att du någon gång behöver opereras för cancer (eller behöver opereras för cancer igen)?

- ☐ 1 Inte alls orolig
- ☐ 2 Inte särskilt orolig
- ☐ 3 Ganska orolig
- ☐ 4 Mycket orolig

Dina erfarenheter av cancergenetisk mottagning

Nedan får du ett par öppna frågor som handlar om din kontakt med den cancergenetiska mottagningen. Vi vill att du ärligt delar med dig av dina tankar och upplevelser av att delta i en familjeutredning vid mottagningen.

GC01. Hur upplevde du att du blev bemött av personalen vid mottagningen?

***

GC02. Hur upplevde du informationen du fick?

GC03. Har du några förslag på hur vår verksamhet skulle kunna förbättras?

GC04. Om du vill tillägga något som vi inte har frågat om, vänligen skriv det här nedan:

____

____

____

____

____

____

***

GC05. Om något är oklart i formuläret ovan, till exempel om vi märker att vi ställt en otydlig fråga, får vi kontakta dig för förtydliganden då?

☐ Nej ☐ Ja

⇒ Vilket telefonnummer kan vi i så fall nå dig på?

____

***

a. Kort information om dig

Slutligen ber vi dig besara en kort fråga om dig själv.

b. Vilken högsta utbildning har du? (obligatorisk fråga)

☐ Mindre än 9 års skolgång
☐ Avslutad grundskola eller motsvarande 9 års skolgång
☐ Avslutat gymnasium eller motsvarande 12 års skolgång
☐ Utbildning minst 1 år utöver gymnasium
☐ Examen från universitet eller högskola

***

Tack för att du tog dig tid
att dela med dig av dina upplevelser.

Du är välkommen att hör av dig till oss
om du har några frågor eller funderingar kring studien.

DIRECT-studien

Direct information to at-risk relatives – A randomized controlled trial

Studietelefon: 076-69 60 648
E-mail: directstudien.rct@umu.se
Ansvarig forskare: Anna Rosén
Institutionen för Strålningsvetenskaper
Umeå universitet
SE-901 87 Umeå

UMEÅ UNIVERSITET

Hej!

Du har tidigare samtyckt till att delta i en forskningsstudie som kallas DIRECT, och vi ber dig besvara detta frågeformulär.

Vi genomför studien för att öka kunskapen om individers upplevelse att få kännedom om en ärftlig förhöjd risk för cancer. Vi har tidigare bett dig besvara samma frågor, men för att kunna studera förändring i välbefinnande över tid ber vi dig besvara samma frågor en gång till.

Det tar runt 10 minuter att svara beroende på hur mycket fri text du skriver.

Dina svar kommer att behandlas anonymt och säkert. Endast behöriga forskare kommer att ta del av datat. Analyser och rapportering görs på gruppnivå.

Alla personuppgifter hanteras enligt bestämmelser i dataskydds-förordningen (GDPR) och personuppgiftslagen (PuL). Du har rätt att få tillgång till, eller rätta, uppgifterna om dig en gång per år genom kontakt med personuppgiftsansvarig vid Umeå universitet. Kontaktuppgifter finns i slutet av formuläret.

Returnera enkäten så här
När du fyllt i alla frågor, postar du formuläret i bifogat svarspostkuvert. Inget frimärke behövs. Om du tappat bort kuvertet så är adressen;

Norrlands universitetssjukhus
DIRECT-studien
Cancergenetisk mottagning
Regionalt cancercentrum Norr
Byggnad 5B, plan 0
90185 Umeå

RAND-36 Hälsa och livskvalitet

RAND-36 handlar om din hälsa och funktion i vardagen. Välj det svarsalternativ som stämmer bäst för dig på varje fråga.

	Utmärkt	Mycket god	God	Någorlunda	Dålig
1. I allmänhet, skulle du säga att din hälsa är:	☐	☐	☐	☐	☐

	Mycket bättre	Något bättre	Ungefär densamma	Något sämre	Mycket sämre
2. **Jämfört med för ett år sedan,** hur skulle du bedöma din hälsa **nu**?	☐	☐	☐	☐	☐

Följande frågor handlar om aktiviteter du kan tänkas ägna dig åt en vanlig dag. **Begränsar din nuvarande hälsa dig** i dessa aktiviteter? Om ja, hur mycket?

	Ja, mycket begränsad	Ja, lite begränsad	Nej, inte alls begränsad
3. **Fysiskt ansträngande aktiviteter**, t.ex. löpning, lyfta tunga föremål, delta i ansträngande idrotter	☐	☐	☐
4. **Måttligt ansträngande aktiviteter**, t.ex. flytta ett bord, dammsuga, promenera eller cykla	☐	☐	☐
5. Lyfta eller bära matkassar	☐	☐	☐
6. Gå upp för **flera** trappor	☐	☐	☐
7. Gå upp för **en** trappa	☐	☐	☐
8. Böja dig eller gå ner på knä	☐	☐	☐
9. Gå **mer än ett par kilometer**	☐	☐	☐
10. Gå **flera kvarter** (flera hundra meter)	☐	☐	☐
11. Gå **ett kvarter** (hundra meter)	☐	☐	☐
12. Bada/duscha eller klä på dig	☐	☐	☐

Under de **senaste 4 veckorna**, har du haft något av följande problem med ditt arbete eller andra vanliga dagliga aktiviteter **på grund av din fysiska hälsa**?

	Ja	Nej
13. Dragit ner på **tiden** du ägnat åt arbete eller andra aktiviteter	☐	☐
14. **Fått mindre gjort** än du skulle vilja	☐	☐
15. Begränsats i **vissa** arbetsuppgifter eller andra aktiviteter	☐	☐
16. Haft **svårt** att utföra arbete eller andra aktiviteter (t.ex. det krävdes mer ansträngning)	☐	☐

Under de **senaste 4 veckorna**, har du haft något av följande problem med ditt arbete eller andra vanliga dagliga aktiviteter **på grund av känslomässiga problem** (t.ex. att du känt dig nere eller orolig)?

	Ja	Nej
17. Dragit ner på **tiden** du ägnat åt arbete eller andra aktiviteter	☐	☐
18. **Fått mindre gjort** än du skulle vilja	☐	☐
19. Utfört arbete eller andra aktiviteter mindre **noggrant** än vanligt	☐	☐

RAND Corporation, USA, äger upphovsrätt till originalet, som togs fram inom Medical Outcomes Study.
Registercentrum Sydost (RCSO) distribuerar översättningen av RAND-36, svensk version 2013-05-21.

	Inte alls	Lite grand	Måttligt	Ganska mycket	Extremt mycket
20. Under de **senaste 4 veckorna**, i vilken omfattning har din fysiska hälsa eller känslomässiga problem stört dina vanliga sociala aktiviteter med familj, släkt, vänner, grannar eller föreningar etc.?	☐	☐	☐	☐	☐

	Ingen	Mycket lätt	Lätt	Måttlig	Svår	Mycket svår
21. Hur mycket **fysisk** smärta har du haft under de **senaste 4 veckorna**?	☐	☐	☐	☐	☐	☐

	Inte alls	Lite grand	Måttligt	Ganska mycket	Extremt mycket
22. Under de **senaste 4 veckorna**, hur mycket har smärta stört ditt vanliga arbete (gäller både arbete utanför hemmet och hushållsarbete)?	☐	☐	☐	☐	☐

Följande frågor handlar om hur du känner dig och hur det har varit **under de senaste 4 veckorna**. Ange det svar som stämmer bäst med hur du känt dig.

Hur mycket av tiden under de **senaste 4 veckorna** ...	Hela tiden	Största delen av tiden	En stor del av tiden	En viss del av tiden	En liten del av tiden	Inget av tiden
23. Har du känt dig pigg?	☐	☐	☐	☐	☐	☐
24. Har du känt dig mycket nervös?	☐	☐	☐	☐	☐	☐
25. Har du känt dig så nere att ingenting kunnat muntra upp dig?	☐	☐	☐	☐	☐	☐
26. Har du känt dig lugn och harmonisk?	☐	☐	☐	☐	☐	☐
27. Har du känt dig energisk?	☐	☐	☐	☐	☐	☐
28. Har du känt dig dyster och ledsen?	☐	☐	☐	☐	☐	☐
29. Har du känt dig utsliten?	☐	☐	☐	☐	☐	☐
30. Har du känt dig lycklig?	☐	☐	☐	☐	☐	☐
31. Har du känt dig trött?	☐	☐	☐	☐	☐	☐

	Hela tiden	Största delen av tiden	En viss del av tiden	En liten del av tiden	Inget av tiden
32. Under de **senaste 4 veckorna**, hur mycket av tiden har din **fysiska hälsa eller känslomässiga problem** stört dina sociala aktiviteter (som att träffa vänner, släktingar etc.)?	☐	☐	☐	☐	☐

Hur väl stämmer följande påståenden in på dig?

	Stämmer helt	Stämmer ganska bra	Vet inte	Stämmer ganska dåligt	Stämmer inte alls
33. Jag verkar ha något lättare att bli sjuk än andra människor	☐	☐	☐	☐	☐
34. Jag är lika frisk som andra jag känner	☐	☐	☐	☐	☐
35. Jag tror att min hälsa kommer att försämras	☐	☐	☐	☐	☐
36. Min hälsa är utmärkt	☐	☐	☐	☐	☐

RAND Corporation, USA, äger upphovsrätt till originalet, som togs fram inom Medical Outcomes Study.
Registercentrum Sydost (RCSO) distribuerar översättningen av RAND-36, svensk version 2013-05-21.

Nr: _ _ _

STAI- S

Instruktioner: Här nedan följer några påståenden som beskriver hur man kan känna sig. Läs varje påstående noga och ringa sedan in den siffra som bäst beskriver hur du känner dig just nu, i detta ögonblick. Det finns inga "rätt" eller "fel". Använd inte för mycket tid för varje påstående utan svara utifrån det du tycker bäst beskriver dina nuvarande känslor.

Siffrorna har följande betydelse:

1 = Inte alls, 2 = Något, 3 = En hel del, 4 = Väldigt mycket.

1. Jag känner mig lugn	1	2	3	4
2. Jag känner mig trygg	1	2	3	4
3. Jag är spänd	1	2	3	4
4. Jag är ångestfylld	1	2	3	4
5. Jag känner mig väl till mods	1	2	3	4
6. Jag känner mig upprörd	1	2	3	4
7. Jag oroar mig över tänkbara olyckor	1	2	3	4
8. Jag känner mig utvilad	1	2	3	4
9. Jag känner mig ängslig	1	2	3	4
10. Jag känner välbefinnande	1	2	3	4
11. Jag känner mig självsäker	1	2	3	4
12. Jag känner mig nervös	1	2	3	4
13. Jag är uppjagad	1	2	3	4
14. Jag känner mig överkänslig	1	2	3	4
15. Jag är avslappnad	1	2	3	4
16. Jag känner mig belåten	1	2	3	4
17. Jag är orolig	1	2	3	4
18. Jag känner mig överspänd och "skakis"	1	2	3	4
19. Jag känner mig glad	1	2	3	4
20. Jag känner mig angenäm	1	2	3	4

STAI-T

Instruktioner: Här nedan följer några påståenden som beskriver hur man kan känna sig. Läs varje påstående noga och ringa sedan in den siffra som bäst beskriver hur du vanligen känner dig. Det finns inga "rätt" eller "fel". Använd inte för mycket tid för varje påstående utan svara det som du tycker bäst beskriver hur du vanligen känner dig.

Siffrorna har följande betydelse:

1 = Nästan aldrig, 2 = Ibland, 3 = Ofta, 4 = Nästan alltid.

1. Jag känner mig angenäm	1	2	3	4
2. Jag blir fort trött	1	2	3	4
3. Jag känner mig gråtfärdig	1	2	3	4
4. Jag önskar jag kunde vara lika lycklig som andra verkar vara	1	2	3	4
5. Jag går miste om saker för att jag inte kan bestämma mig fort nog	1	2	3	4
6. Jag känner mig utvilad	1	2	3	4
7. Jag är "lugn och samlad"	1	2	3	4
8. Jag känner att svårigheter växer sig så stora att jag inte klarar av dem	1	2	3	4
9. Jag oroar mig för mycket över saker som egentligen inte spelar någon roll	1	2	3	4
10. Jag är lycklig	1	2	3	4
11. Jag är benägen att ta saker och ting för allvarligt	1	2	3	4
12. Jag saknar självförtroende	1	2	3	4
13. Jag känner mig trygg	1	2	3	4
14. Jag försöker undvika att ta itu med svårigheter och kriser	1	2	3	4
15. Jag känner mig nedstämd	1	2	3	4
16. Jag är belåten	1	2	3	4
17. Någon oväsentlig tanke far genom mitt huvud och stör mig	1	2	3	4
18. Jag tar besvikelser så hårt att jag ej kan släppa dem ur mina tankar	1	2	3	4
19. Jag är en stabil person	1	2	3	4
20. Jag blir spänd och upprörd när jag tänker på mina aktuella bekymmer	1	2	3	4

Instrument för kvalitativ uppföljning av forskningspersoners upplevelser (STAI, 2 av 2 sidor).
Referens: *Spielberger, C.D., Gorsuch, R.L., & Lushene, R.E. (1970). The State-Trait Anxiety Inventory: Testmanual. Palo Alto, CA: Consulting Psychologist Press*

Cancer Worry Scale

Adapted from Lerman C, 1994, Douma KH, 2010, Lammens CRM, 2010 and translated to Swedish.

Nu följer ett antal frågor om dina tankar och känslor kring cancer. Frågorna gäller oavsett om du har cancer eller inte.

Hur ofta har du tänkt på risken att utveckla cancer (eller att utveckla cancer igen)?

- ☐ Sällan eller aldrig
- ☐ Ibland
- ☐ Ofta
- ☐ Nästan hela tiden

Har dessa tankar påverkat ditt humör?

- ☐ Inte alls
- ☐ Lite
- ☐ En del
- ☐ Mycket

Har dessa tankar påverkat din förmåga att utföra dagliga sysslor?

- ☐ Inte alls
- ☐ Lite
- ☐ En del
- ☐ Mycket

Hur orolig är du över risken att någon gång utveckla cancer (eller att utveckla cancer igen)?

- ☐ Inte alls orolig
- ☐ Inte särskilt orolig
- ☐ Ganska orolig
- ☐ Mycket orolig

Hur ofta oroar du dig över att utveckla cancer (eller att utveckla cancer igen)?

- ☐ Sällan eller aldrig
- ☐ Ibland
- ☐ Ofta
- ☐ Nästan hela tiden

Hur stort problem är denna oro?

☐ Mycket litet
☐ Ganska litet
☐ Ganska stort
☐ Mycket stort

Hur ofta oroar du dig för risken att dina familjemedlemmar kommer att utveckla cancer?

☐ Sällan eller aldrig
☐ Ibland
☐ Ofta
☐ Nästan hela tiden

Hur orolig är du över möjligheten att du någon gång behöver opereras för cancer (eller behöver opereras för cancer igen)?

☐ Inte alls orolig
☐ Inte särskilt orolig
☐ Ganska orolig
☐ Mycket orolig

Om du vill tillägga något som vi inte har frågat om, vänligen skriv det här nedan:

Tack för att du tog dig tid att besvara formuläret.

Du är välkommen att höra av dig till oss
om du har några frågor eller funderingar kring studien.

DIRECT-studien

Direct information to at-risk relatives – A randomized controlled trial

Studietelefon: 076-69 60 648
E-mail: directstudien.rct@umu.se
Ansvarig forskare: Anna Rosén
Institutionen för strålningsvetenskaper
Umeå universitet
SE-901 87 Umeå

UMEÅ UNIVERSITET
